# Lineage-specific positive selection at the merozoite surface protein 1 (*msp1*) locus of *Plasmodium vivax *and related simian malaria parasites

**DOI:** 10.1186/1471-2148-10-52

**Published:** 2010-02-19

**Authors:** Hiromi Sawai, Hiroto Otani, Nobuko Arisue, Nirianne Palacpac, Leonardo de Oliveira Martins, Sisira Pathirana, Shiroma Handunnetti, Satoru Kawai, Hirohisa Kishino, Toshihiro Horii, Kazuyuki Tanabe

**Affiliations:** 1 Laboratory of Malariology, Research Institute for Microbial Diseases, Osaka University, Osaka 565-0871, Japan; 2 Department of Molecular Protozoology, Research Institute for Microbial Diseases, Osaka University, Osaka 565-0871, Japan; 3 Bioinformatics and Molecular Evolution, University of Vigo, Vigo 36310, Spain; 4 Malaria Research Unit, Department of Parasitology, Faculty of Medicine, University of Colombo, Colombo, Sri Lanka; 5 Institute of Biochemistry, Molecular Biology and Biotechnology, University of Colombo, Colombo, Sri Lanka; 6 Center for Tropical Medicine and Parasitology, Dokkyo Medical University, Tochigi 321-0293, Japan; 7 Graduate School of Agriculture and Life Sciences, University of Tokyo, Tokyo 113-8657, Japan

## Abstract

**Background:**

The 200 kDa merozoite surface protein 1 (MSP-1) of malaria parasites, a strong vaccine candidate, plays a key role during erythrocyte invasion and is a target of host protective immune response. *Plasmodium vivax*, the most widespread human malaria parasite, is closely related to parasites that infect Asian Old World monkeys, and has been considered to have become a parasite of man by host switch from a macaque malaria parasite. Several Asian monkey parasites have a range of natural hosts. The same parasite species shows different disease manifestations among host species. This suggests that host immune responses to *P. vivax*-related malaria parasites greatly differ among host species (albeit other factors). It is thus tempting to invoke that a major immune target parasite protein such as MSP-1 underwent unique evolution, depending on parasite species that exhibit difference in host range and host specificity.

**Results:**

We performed comparative phylogenetic and population genetic analyses of the gene encoding MSP-1 (*msp1*) from *P. vivax *and nine *P. vivax*-related simian malaria parasites. The inferred phylogenetic tree of *msp1 *significantly differed from that of the mitochondrial genome, with a striking displacement of *P. vivax *from a position close to *P. cynomolgi *in the mitochondrial genome tree to an outlier of Asian monkey parasites. Importantly, positive selection was inferred for two ancestral branches, one leading to *P. inui *and *P. hylobati *and the other leading to *P. vivax*, *P. fieldi *and *P. cynomolgi*. This ancestral positive selection was estimated to have occurred three to six million years ago, coinciding with the period of radiation of Asian macaques. Comparisons of *msp1 *polymorphisms between *P. vivax*, *P. inui *and *P. cynomolgi *revealed that while some positively selected amino acid sites or regions are shared by these parasites, amino acid changes greatly differ, suggesting that diversifying selection is acting species-specifically on *msp1*.

**Conclusions:**

The present results indicate that the *msp1 *locus of *P. vivax *and related parasite species has lineage-specific unique evolutionary history with positive selection. *P. vivax *and related simian malaria parasites offer an interesting system toward understanding host species-dependent adaptive evolution of immune-target surface antigen genes such as *msp1*.

## Background

Malaria, a parasitic infection transmitted by the anopheline mosquito, is caused by the genus *Plasmodium*. Of the five parasite species that infect humans, *Plasmodium vivax *is arguably the most prevalent in South and Central America, Melanesia, Asia and the Middle East, accounting for 80 - 90 million cases annually [1]. Unlike *P. falciparum*, which often causes severe malaria, *P. vivax *infections are rarely lethal but a major cause of morbidity in endemic countries. An ideal control strategy is the development of effective vaccines, with potential targets in the asexual blood stage responsible for the clinical manifestation of the disease. The 200 kDa merozoite surface protein 1 (MSP-1), a vaccine candidate antigen that is abundantly expressed on the surface of merozoites and plays critical role in erythrocyte invasion, is one of the major targets of the host immune response [2]. MSP-1 is conserved in all *Plasmodium *species. Disruption of the *Plasmoium *MSP-1 gene has been demonstrated to have a deleterious effect on the parasite growth in experimental animals [3].

The 200 kDa precursor of *P. falciparum *MSP-1 undergoes proteolytic processing, producing four major polypeptides of approximately 83, 30, 38 and 42 kDa from the N-terminus to C-terminus [2]. These polypeptides are on the merozoite surface as a non-covalently associated complex. Coincident with erythrocyte invasion the C-terminal 42 kDa is further cleaved to produce the N-terminal 33 kDa and C-terminal 19 kDa, and except for the latter, all processed fragments are shed [4]. The 19 kDa C-terminal, which contains two epidermal growth factor (EGF)-like domains, remains anchored to the merozoite membrane and is carried into the invaded erythrocytes. These processing events appear to be conserved in *P. knowlesi*, a *P. vivax*-related simian malaria parasite [5]. Both the 42 kDa and 19 kDa polypeptides are considered to be promising vaccine candidates for *P. falciparum *and *P. vivax *[6,7]. However, the gene encoding MSP-1 (*msp1*), which is present as a single copy per haploid parasite genome, is highly polymorphic [8] and thus, presents a major obstacle to effective vaccine development. There is evidence showing that allelic diversity of MSP-1 is strongly associated with strain-specific protective immunity in experimental animals [9,10]. Sequence variation in *msp1 *has previously been studied for *P. falciparum *[8,11,12]. A large proportion of *P. falciparum msp1 *has a dimorphic structure, with alleles classified as belonging to either K1 or MAD20 type. Divergence between these dimorphic alleles is estimated to have occurred approximately 27 - 35 million years ago (mya) [13,14]. Although the biological significance of allelic dimorphism in *P. falciparum msp1 *is unknown, it is likely that K1 and MAD20 alleles are maintained by balancing selection as part of the parasite immune evasion mechanism. We have shown that *P. vivax msp1 *also shows extensive allelic variation but that the polymorphism pattern is clearly different from the dimorphic nature of *P. falciparum msp1 *[15]. Furthermore, in contrast to the presumed ancient origin of *P. falciparum msp1*, the origin of *P. vivax msp1 *polymorphism is relatively recent [16].

*P. vivax *has a unique evolutionary history as compared with *P. falciparum*. It is believed that *P. falciparum *and the closely related chimpanzee parasite, *P. reichenowi*, co-diverged alongside that of humans and chimpanzees [17,18]. In contrast, *P. vivax *is closely related to parasites such as *P. knowlesi *and *P. cynomolgi *that infect Asian Old World monkeys (OWMs), though *P. vivax *does not infect the OWMs [19]. *P. vivax *has recently been proposed to have become a parasite of man by host switch from a macaque malaria parasite sometime between 50,000 to 300,000 years ago [20,21] or 460,000 years ago [22]. *P. hylobati*, a parasite of gibbons in Southeast Asia, is also considered to have originated by host switch from a macaque parasite. Several Asian macaque malaria parasites have a range of natural hosts, e.g., *P. inui *and *P. cynomolgi *infect multiple species of the genera *Macaca *and *Presbytis *[19]. *P. knowlesi *infects not only macaques, leaf monkeys and langurs but also has recently been recognized as the fifth human malaria parasite based from studies in Southeast Asia [23]. Experimental infections of the rhesus macaque, *M. mulatta*, and the Japanese macaque, *M. fuscata*, by *P. coatneyi, P. knowlesi, P. cynomolgi *or *P. inui *result in acute manifestations of malaria with high parasitemia, whereas these parasites produce less symptoms and low-grade infections in the long-tailed or crab eating macaque, *M. fascicularis *[19,24,25]. This indicates the same parasite species causes different disease manifestations among host species. Such differential disease manifestations may suggest that immune responses to *P. vivax*-related malaria parasites greatly differ among host species (although other factors, such as the ability of the parasites to infect a wider range of red blood cells, e.g., reticulocytes vs. mature red cells, can also contribute to virulence). It is thus tempting to invoke that a major immune target parasite protein gene such as *msp1 *underwent unique evolution, depending on parasite species that exhibit difference in host range and host specificity. As such, *P. vivax *and *P. vivax*-related simian malaria parasites are invaluable resources in our attempts to understand adaptive evolution in malaria parasites.

In this study, we investigate (i) the occurrence of natural selection on *msp1 *in lineages of *P. vivax *and related simian malaria parasites through comparative analysis of phylogenetic trees between *msp1 *and the mitochondrial genome, and (ii) using comparative analysis of *msp1 *polymorphism between *P. vivax*, *P. inui *and *P. cynomolgi*, we examine the species-specific diversifying selection on *msp1*. Results obtained here show that *msp1 *evolved under positive selection in ancestral lineages including the one leading to *P. vivax *and its closely related simian parasite species, three to six mya, coinciding with the period of radiation of Asian macaques. Also, a signature of species-specific diversifying selection was detected in *P. vivax*, *P. inui *and *P. cynomolgi*.

## Results

### Divergence of *msp1 *sequence

We obtained 20 new *msp1 *sequences from *P. knowlesi*, *P. inui*, *P. cynomolgi*, *P. fieldi*, *P. fragile *and *P. gonderi *(Additional file 1). An alignment of the sequences, together with previously published sequences including *P. vivax*, *P. simiovale*, *P. hylobati *and *P. coatneyi*, revealed considerable sequence variations between species with substantial sequence length difference between 5097 bp to 5787 bp (Additional file 2). These variations include numerous substitutions, extensive indels and varying numbers of repeats (Additional file 3). Since indels and repeats were unreliably aligned, these were excluded, leaving 4176 bp aligned positions that were used for further analysis (Additional file 3). Nucleotide divergence in the aligned 4176 bp sequences and amino acid divergence were 39% - 42% and 41% - 47%, respectively, between *P. gonderi*, an African OWM parasite and *P. vivax *and related simian malaria parasites. Between *P. vivax *and the seven Asian monkey parasite species divergence was 15% - 21% and 24% - 36% at the nucleotide and amino acid levels, respectively.

### Phylogenetic analysis of the mitochondrial genome and *msp1*

**Figure 1:**
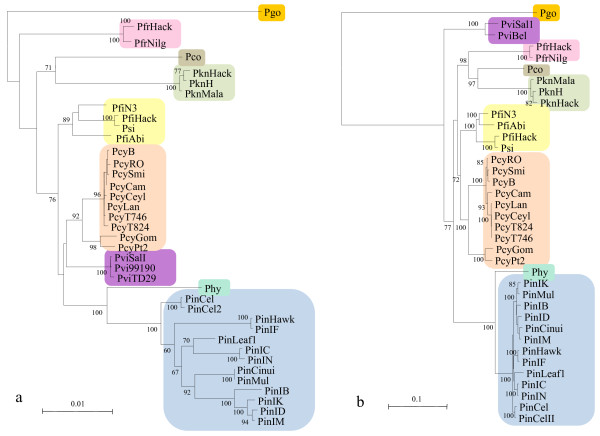
**Phylogenetic trees of the mitochondrial genome and *msp1 *of *P. vivax *and *P. vivax*-related simian malaria parasite species**. (a) The maximum-likelihood (ML) tree of the mitochondrial genome. Aligned sequences of the mitochondrial genome (5818 bp) from *P. vivax *and related simian malaria parasite species were used for constructing the tree with 100 heuristic replicates under the GTR + I + G model with an α = 0.812. (b) The ML tree of the *msp1 *gene. The aligned 4176 bp (Additional file 3) sequences were used for constructing the tree with 100 heuristic replicates under the GTR + I + G model with an α = 0.866. Abbreviations of species and strains are: Pgo = *P. gonderi*, Pfr = *P. fragile*, Pco = *P. coatneyi*, Pkn = *P. knowlesi*, Pfi = *P. fieldi*, Psi = *P. simiovale*, Pcy = *P. cynomolgi*, Pvi = *P. vivax*, Phy = *P. hylobati*, and Pin = *P. inui*; and Hack = Hackeri, Nilg = Nilgiri, Mala = Malayan, N3 = N-3, Abi = A.b.introlatus, Smi = Smithsonian, Cam = Cambodian, Ceyl = Ceylonensis, Lan = Langur, Gom = Gombak, SalI = Sal-I, Bel = Belem, Cel = Celebes, CelII = Celebes II, Hawk = Hawking, Leaf1 = Leaf monkey #1, Mul = Mulligan. In both (a) and (b), only bootstrap values = 60% are indicated at nodes.

The mitochondrial genome of *Plasmodium *has been shown to be free from strong positive selection [20,26]. In this work, we obtained 28 new sequences of the mitochondrial genomes from *P. knowlesi*, *P. fragile*, *P. inui*, *P. cynomolgi *and *P. fieldi*. Together with reported sequences, we constructed the Maximum likelihood (ML) tree of the mitochondrial genome of *P. vivax *and related simian parasites and compared the tree with that constructed from *msp1*. The mitochondrial ML tree revealed two major clades using *P. gonderi *as an outgroup: one for a clade of *P. coatneyi *and *P. knowlesi*, and the other for a clade of *P. fieldi*, *P. cynomolgi*, *P. vivax*, *P. hylobati *and *P. inui *(Figure 1a). (*P. simiovale *was grouped into the *P. fieldi *lineage in this study, as noted in Methods.) Relationship between these two clades and *P. fragile *was not well resolved. These results are basically consistent with the tree topologies constructed using two nuclear genes, *β-tubulin *and *CDC-2*, and the plastid gene *TufA *[21], 18S rRNA gene [27], and the mitochondrial cytb gene [28]. A ML tree constructed using sequences of three mitochondrial protein coding regions was similar to the ML tree using the whole mitochondrial genome sequence (not shown).

The ML tree of *msp1 *shows two major clades using *P. gonderi *as an outgroup, one for *P. vivax *and the other for the seven Asian monkey parasite species. In the latter clade, three sub-clades were noted: (i) *P. fragile*, *P. coatneyi *and *P. knowlesi*, (ii) *P. fieldi *and *P. cynomolgi*, and (iii) *P. hylobati *and *P. inui *(Figure 1b). Topologies of the mitochondrial tree and the *msp1 *tree appear to be distinct, with striking difference in that *P. vivax *was displaced from its position closest to *P. cynomolgi *in the mitochondrial tree to an outlier of the seven Asian monkey parasites in the *msp1 *tree. Applying the mitochondrial genome sequence data to the *msp1 *topology, significant difference was noted between the two trees (Δ*li *= -42.1, p = 0.003) (Table 1). Similarly, applying the *msp1 *sequence data to the mitochondrial genome topology, the two trees were significantly different (Δ*li *= -23.6, p = 0.041). Additionally, *msp1 *alignment columns were separated into a partition composed of first and second codon position sites (partition of nonsynonymous sites; codon 1 + 2) and another partition comprised of third codon position sites (partition of synonymous sites; codon 3), with trees constructed independently for the two distinct partitions. The topology of the ML tree for *msp1 *codon 1 + 2 was the same as that of the *msp1 *ML tree using all sites, whereas it significantly differed from the topology of *msp1 *ML tree of codon 3 (Δ*li *= -23.4, p = 0.030). Applying the mitochondrial genome sequence data to the topology of the *msp1 *codon 3 tree, the difference was not significant between the two topologies (Δ*li *= -9.4, p = 0.333). These results indicate that the *msp1 *best tree is significantly different from the mitochondrial genome best tree, and that the difference between the mitochondrial genome best tree and the *msp1 *best tree is largely due to nonsynonymous substitutions in the latter, suggesting positive selection in the evolution of *msp1*.

**Table 1:** Topology difference between the mitochondrial genome tree and *msp1 *trees.

		Mitochondrial genome^a^		*msp1*^b^	
		
	Topology of the ML best tree	Δ*li*^c^	p-value^d^	Δ*li*^c^	p-value^d^
mitochondrial genome	(((((Pcy,Pvi),(Phy,Pin)),Pfi),(Pco,Pkn)),Pfr,Pgo)	(-14380.5)		-23.6	0.041
*msp1 *all sites *msp1 *codon 1 + 2	((((Pcy,Pfi),((Pco,Pkn),Pfr)),(Phy,Pin)),Pvi,Pgo) ((((Pcy,Pfi),((Pco,Pkn),Pfr)),(Phy,Pin)),Pvi,Pgo)	-42.1 -42.1	0.003 0.003	(-28818.3) (-28818.3)	
*msp1 *codon 3	((((Pcy,(Pfi,Pvi)),(Phy,Pin)),(Pco,Pkn)),Pfr,Pgo)	-9.4	0.333	-23.4	0.030

^a ^The mitochondrial genome sequences were applied to each of the 3 *msp1 *trees (*msp1 *all sites, *msp1 *codon 1 + 2, and *msp1 *codon 3), and topology difference was evaluated using the PAML software package [52].

^b ^The *msp1 *sequences were applied to each of the mitochondrial genome tree and the *msp1 *codon 3 tree, and topology difference was evaluated.

^c ^Log-likelihood difference of the alternative tree against the best tree.

^d ^p-values are obtained with the Shimodaira-Hasegawa test [51].

### Lineage-specific positive selection in *msp1*

**Figure 2:**
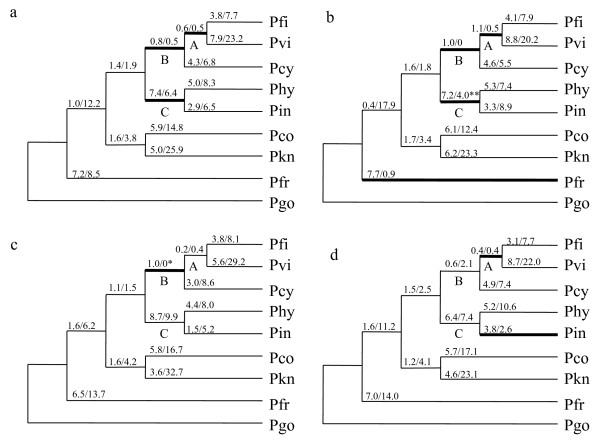
**Lineage-specific positive selection in the phylogeny of *msp1 *from *P. vivax *and *P. vivax*-related simian malaria parasite species**. The maximum-likelihood (ML) tree topology was constructed using sequences of *msp1 *codon 3 for (a) the whole *msp1 *gene, (b) the 5' region, (c) the central region and (d) the 3' region. The estimates of kA and kS (×100) are shown above each branch. Branches showing ω (= kA/kS) >1 are bold-lined. A branch showing ω significantly greater than 1 (P < 0.05), when the free ratio model was compared with the constrained model that fixed ω of the branch of interest at 1, is double-asterisked; and that weakly higher than 1 (P < 0.1) is asterisked. Abbreviations are: Pfi, *P. fieldi *N-3; Pvi, *P. vivax *Sal-I; Pcy, *P. cynomolgi *Smithsonian; Phy, *P. hylobati*; Pin, *P. inui *Celebes; Pco, *P. coatneyi*; Pkn, *P. knowlesi *H; Pfr, *P. fragile *NIH; Pgo, *P. gonderi*.

We examined the occurrence of lineage-specific positive selection using the free-ratio model, which allows variable ω (= kA/kS) among branches. For this analysis, the ML topology of the *msp1 *third codon positions was used. The free-ratio model revealed three specific branches having ω > 1: the branch ancestral to *P. fieldi *and *P. vivax *(branch A, ω_1_), the branch ancestral to branch A and *P. cynomolgi *(branch B, ω_2_), and the branch ancestral to *P. hylobari *and *P. inui *(branch C, ω_3_) (Figure 2a). Values of ω_1_, ω_2 _and ω_3 _were not significantly greater than one by likelihood ratio test (LRT) when the free model was compared with the constrained models that fixed ω_1_, ω_2 _or ω_3 _at one. Since it is likely that a large number of sites in *msp1 *may be under strong purifying selection due to constraints of the protein's function, we estimated ω values for three separate *msp1 *regions: the 5' region (1851 bp), the central region (1227 bp) and the 3' region (1098 bp); corresponding to the N-terminal 83 kDa fragment, the central 33 kDa + 38 kDa fragments, and the C-terminal 42 kDa fragment, respectively. In the 5' region, four branches showed ω > 1: the lineage to *P. fragile*, branches A, B and C. Among these, ω of branch C was significantly greater than one when the free model was compared with the constrained model that fixed the ω at one (p = 0.041 by LRT test) (Figure 2b). In the central region, ω > 1 was detected in branch B (Figure 2c). When the free model was compared with the constrained model that fixed the ω at one, ω was slightly higher than one though not significant (p = 0.076). Two branches (a lineage leading to *P. inui *and branch B) in the 3' region, likewise, showed ω > 1 but were not significant (Figure 2d). As a whole, we observe that positive selection was acting on the *msp1 *5' region and central region at the early period after the divergence among *P. fieldi*, *P. vivax*, *P. cynomolgi*, *P. hylobati*, and *P. inui*.

To infer the time of the two branches where positive selection was detected (branches B and C in Figure 2), we estimated the divergence time of lineages of interest using the mitochondrial sequences. The relative rate tests revealed evolutionary rate constancy in two different groups with rate heterogeneity between them (Additional file 4): one group (Group I) includes *P. gonderi*, *P. vivax*, *P. cynomolgi *and *P. fieldi *using *P. chabaudi*, a rodent malaria parasite, as an outgroup, and the other (Group II) includes *P. knowlesi*, *P. hylobati *and *P. inui *using *P. gonderi *as an outgroup. In Group I, the average pairwise genetic distance between *P. gonderi *and *P. fieldi*/*P. vivax*/*P. cynomolgi *was 0.036 ± 0.003 and that between *P. fieldi *and *P. vivax*/*P. cynomolgi *was 0.011 ± 0.001. Assuming that *P. gonderi *and Asian monkey parasite species diverged around 10 mya along with their respective hosts [18], the divergence time of *P. fieldi*, *P. vivax *and *P. cynomolgi *was 3.0 ± 0.3 mya. In Group II, the distance between *P. knowlesi *and *P. hylobati*/*P. inui *was 0.035 ± 0.002 and that between *P. hylobati *and *P. inui *was 0.025 ± 0.002. Adopting the divergence time of *P. knowlesi *and other Asian OWM parasites of 6.3 mya [18], the divergence time of *P. hylobati *and *P. inui *was 4.6 ± 0.4 mya. Dating of the common ancestor of branches B and C was not successfully estimated, due to rate heterogeneity between the two groups. We therefore conservatively adopted the divergence time (6.3 ± 1.4 mya) of *P. knowlesi *and other Asian monkey parasites as an older timing of the common ancestor of branches B and C. Thus, branch B was estimated to lie between 3.0 ± 0.3 and 6.3 ± 1.4 mya, and branch C to lie between 4.6 ± 0.4 and 6.3 ± 1.4 mya.

### Polymorphism of *msp1 *sequences

Intraspecific nucleotide diversity (π) varied between 1.9% and 3.4% in *P. vivax*, *P. cynomolgi*, and *P. inui*, in which ten or more sequences were available (Additional file 1). Average pairwise amino acid difference was 3.7% to 5.0%. Our previous analysis has revealed the evolution of *msp1 *polymorphism in *P. vivax *and *P. cynomolgi *after speciation [16]. The present study extends this recent evolution of *msp1 *polymorphism to *P. inui *(Figure 1).

### Detection of the signature of diversifying selection

**Figure 3:**
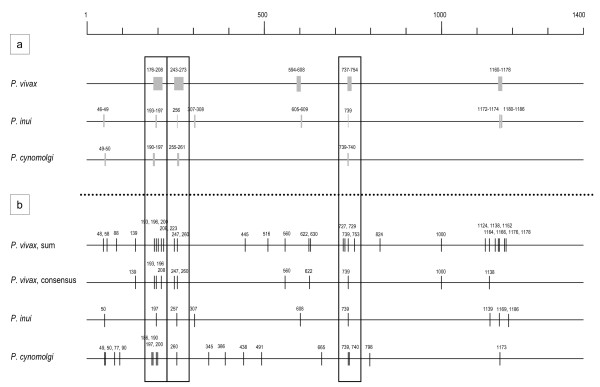
**Positively selected codon sites along the *msp1 *alignment of *P. vivax *and *P. vivax*-related simian malaria parasites**. Positively selected sites with ω (= dN/dS) >1 were detected by (a) omegaMap [29] and (b) HyPhy/Datamonkey [30]. Boxes indicate positively selected regions shared by three parasite species. In (a), positions of codon showing ω > 1 with more than 95% posterior probability are denoted by half-tone bars. In (b), individual sites with ω significantly greater than 1 are mapped by vertical lines. In *P. vivax*, computations were repeated five times after random sampling from a pool of sequences, and sites detected at least once in the analyses; or those detected 3 times or more are shown as "sum" and "consensus", respectively. Refer to Additional file 3; figure S1 for individual amino acid replacements.

To detect amino acid sites under positive selection in *msp1 *sequences, we used the omegaMap to perform a Bayesian inference on ω (= dN/dS) [29]. This method allows us to identify positively selected amino acid sites in sequences even in the presence of widespread recombination breakpoints, thus applicable to *P. vivax msp1 *sequences, in which recombination events have been inferred [15]. The selection parameter ω was analyzed for *msp1 *alignments of *P. vivax*, *P. cynomolgi *and *P. inui*. *P. vivax *showed five regions with posterior probability of positive selection > 95% (Figure 3). *P. inui *and *P. cynomolgi *had eight and four regions, respectively, with posterior probability > 95%. Most of these positively selected regions/sites overlapped among the three species: all five regions in *P. vivax *and all four regions in *P. cynomolgi *coincided with those regions in *P. inui *(Figure 3a), suggesting between-species conservation of positively selected regions/sites. The number of positively selected sites varied among species: 116 sites in *P. vivax*, 28 sites in *P. inui *and 22 sites in *P. cynomolgi *(Additional file 3). We are not certain why *P. vivax *showed numerous positively selected sites, although closer inspection of those positively selected regions revealed longer haplotype blocks in *P. vivax *(Additional file 5). Such haplotype blocks may have falsely produced relatively numerous selection sites. Of note, at positively selected amino acid positions that were shared by the three parasite species, amino acid substitutions were remarkably divergent: e.g. amino acid substitutions at 739 (position 999 in the *P. vivax *Sal-1 sequence in Additional file 3; figure S1) were R/Q/A/T in *P. vivax*, H/Q/T/P/N in *P. inui*, and L/Q/V in *P. cynomolgi*.

Positively selected sites within non-recombination sequence segments were also detected using HyPhy/Datamonkey [30]. Due to the inherent limitation in the maximum number of sequences (n = 14) that can be handled by the recombination detection program, 14 *P. vivax *sequences were randomly selected from a total of 43 sequences and analyzed, with the procedure being repeated five times. The number of positively selected sites varied from nine to 15 in five computations, and those detected thrice or more (referred to as consensus sites) were eleven, while those detected in at least one computation were 29 (Figure 3b). The numbers of positively selected sites were nine and 18 in *P. inui *and *P. cynomolgi*, respectively. Although only one positively selected site (at amino acid position 739) was shared by the three parasite species, there were two additional selected regions/sites that were positioned nearby in each species: at 193 - 200 and 247 - 260. It is also worth noting that in each of the three species, the positively selected sites detected by either omegaMap and HyPhy/Datamonkey roughly overlaps (Figure 3): six of the eleven sites detected by HyPhy in *P. vivax *(consensus); eight of the nine sites in *P. inui*; and nine of the 18 sites in *P. cynomolgi*. Positively selected sites are somewhat clustered in the 5' regions.

## Discussion

### Ancestral positive selection in *P. vivax *and related simian malaria parasites

This study presents evidence of a signature for ancestral positive selection on an immune target surface antigen gene (*msp1*) of malaria parasites. Differences in the phylogenetic trees between the mitochondrial genome and *msp1 *of *P. vivax *and related simian malaria parasites suggest that *msp1s *have evolved under positive selection. Positive selection was detected in an ancestral lineage leading to *P. inui *and *P. hylobati *(branch C) (Figure 2) and weak positive selection in an ancestral branch leading to *P. vivax, P. fieldi*, and *P. cynomolgi *(branch B). In these branches, positive selection was not detected at the whole gene level but in separate *msp1 *regions. It is rare to find a gene showing evidence for positive selection at the whole gene level or a long gene region because most genes are under purifying selection [31], and thus small regions that are under positive selection, if any, may not be readily detectable. We do not therefore exclude the possibility that smaller *msp1 *regions and/or limited amino acid sites were under positive selection in ancestral branches other than branches B and C of *P. vivax*-related simian malaria parasite lineages.

Estimated time frame of these lineages was between 3.0 and 6.3 mya. It should be noted that 6.3 mya is a conservative estimate because of the lack of an appropriate calibration point to estimate a common ancestor of branches B and C. With this in mind, the estimated time frame seems to coincide well with the period of radiation events of Asian macaques (3.7-5.1 mya) [32]. It is plausible that Asian macaque malaria parasites radiated along with radiation events of host monkey species. Natural infections in macaques such as *M. fascicularis *with several Asian simian malaria parasites like *P. cynomolgi *and *P. inui *are relatively benign, whereas experimental infections with these parasites are severe and frequently lethal to non-natural macaque hosts that inhabit areas free of simian malaria [33]. To survive in such partially resistant hosts, parasites would have developed a mechanism(s) to evade host immune attacks and thus favorable mutations were probably accumulated in immune target antigen genes such as *msp1*. During the radiation of macaques in Southeast Asia, sympatric speciation of macaques occurred and this may have provided a niche for a potential expansion of host range of ancestral malaria parasites. Some ancestral parasites that successfully gained favorable mutations for immune evasion in major antigen genes during the radiation period may have succeeded in adapting to newly appeared macaque species, thus leaving a signature for ancestral positive selection on *msp1*.

### Diversifying selection in *P. vivax*, *P. inui *and *P. cynomolgi*

Polymorphisms of the whole *msp1 *sequences or sequences of three separate *msp1 *regions do not show evidence for diversifying selection in *P. vivax *and *P. cynomolgi *[16]. We confirmed this for *P. inui *(data not shown). This is not unexpected because MSP-1 is essential for the parasite survival [2]. However, we obtained a signature for diversifying selection at specific amino acid sites in these parasite species. Interestingly, three positively selected sites/regions were shared by the three parasite species (Figure 3), suggesting a common structural feature of diversifying selection. However, amino acid substitutions in these sites/regions greatly differed among the three parasite species. Also, other positively selected sites/regions were not shared among the three species. These indicate that the evolution of *msp1 *polymorphism is species-specific in *P. vivax *and related simian parasite species. The species-specific feature of diversifying selection on *msp1 *underscores the importance of understanding the protective immune response to the vaccine candidate MSP-1 in a species-specific context.

Little structural information is available for MSP-1, except for the C-terminal 19 kDa polypeptide, which contains cysteine-rich two epidermal growth factor-like domains [34,35]. A hydropathy profile suggests that most of MSP-1 regions, apart from the N-terminal signal peptide and the 19 kDa polypeptide, are hydrophilic (data not shown), being consistent with the prediction that MSP-1 is a component of the merozoite surface coat [36]. It is thus presumed that positively selected sites are antibody-binding epitopes. Amino acid substitutions are mostly radical ones in terms of chemical properties affecting, for example, the acidity/basicity and hydrophobicity/hydrophilicity nature, likely exerting influence in the antibody binding epitopes of the protein. Alternatively, amino acid changes in these positively selected sites may alter the ability of antigen-derived peptides to bind to allelic forms of class II major histocompatibility complex (MHC), resulting in specific loss of an immune recognition by particular T cells.

In *P. vivax*, positively selected sites/regions appears to be clustered in the 5' region of *msp1*. Seroepidemiology studies suggest that the 5' region is subjected to diversifying selection by immune pressures. A high proportion of individuals infected with *P. vivax *in Colombia contain IgG antibodies against Pv200L, a N-terminal protein of *P. vivax *MSP-1 (residues 69 to 430 of the Belem strain). This region also confers partial protection from *P. vivax *challenge infection in *Aotus *monkeys which are experimental models but not natural hosts of *P. vivax *[37]. Clinical protection has, likewise, been correlated to IgG antibodies against the N-terminal part of MSP-1 (residues 170 to 675) in western Brazilian Amazon [38]. Similarly, positively selected amino acid sites were detected in the C-terminal 33 kDa fragment that corresponds to one of the cleaved fragments of the 42 kDa polypeptide of *P. falciparum *MSP-1 [39]. In individuals living in an endemic area in Sri Lanka, naturally acquired antibodies were directed more strongly to the 42 kDa polypeptide of *P. vivax *MSP-1 than to the 19 kDa fragment [40], suggesting stronger immunogenicity of the 33 kDa polypeptide. It is therefore probable that diversifying selection on the 3' region of *P. vivax msp1 *corresponding to the 33 kDa region is driven by host's immune pressure. Although positively selected sites/regions were also detected in the central region, no immunological study has been done to date, thus no clear inference can be made for diversifying selection at this region.

In the present study, highly polymorphic sequence regions consisting of indels and different numbers of tandem repeats with varying repeat units and lengths (Additional file 6) were not analyzed due largely to limitations in reliably aligning sequences. In *P. falciparum msp1*, sequence variation of tandem repeats is confined to two variable blocks, blocks 2 and 8 [11]. In block 2 of *P. falciparum msp1*, different types of tripeptide repeats have been shown to be involved in protective immunity in individuals living in a malaria endemic area [41]. It has been reported that sera of *P. vivax*-infected patients contain IgG antibodies strongly recognizing the N-terminal variable blocks of *P. vivax *MSP-1, whereas they do not react to the N-terminal conserved blocks [42]. Thus, variations in the number and sequence units of tandem repeats in *msp1 *from *P. vivax *and related simian malaria parasites may well be associated with allele-linked protective immunity or strain-specific protective immunity.

## Conclusion

The present study revealed a signature for lineage-specific ancestral positive selection on *msp1 *in *P. vivax *and related simian malaria parasites. This ancestral positive selection is inferred to have occurred three to six mya, coinciding with the period of the radiation of Asian macaques. Ancestral Asian macaque parasites may have succeeded in expanding their host ranges when parasites successfully generated adaptive evolution in immune target antigen genes such as *msp1*. The recently published parasite genomes of *P. vivax *and *P. knowlesi*, [43,44] have revealed that the related two species evolved unique genetic systems of antigenic variation (such as *vir *family in *P. vivax *and *SICAvar*- and *kir*-families in *P. knowlesi*) for evading host immune responses. Analysis of ancestral positive selection would unveil what is essential for successful adaptation to new hosts, and eventually help to further understanding the evolutionary arms race between malaria parasites and their hosts. Additionally, in this study, a signature for species-specific diversifying selection on *msp1 *was detected in *P. vivax, P. inui *and *P. cynomolgi*, underscoring the importance of understanding the protective immune response to the vaccine candidate MSP-1 in a species-specific context.

## Methods

### *Plasmodium *species sequenced

Sequences of *msp1 *and the mitochondrial genome of *P. vivax*-related simian malaria parasite species were obtained from the following *Plasmodium *species (and strains): *P. gonderi*, *P. fragile *(two strains: Hackeri and Nilgiri), *P. knowlesi *(three strains: Malayan, Hackeri and H), *P. coatneyi*, *P. hylobati*, *P. simiovale*, *P. fieldi *(three strains: N-3, A. b. introlatus and Hackeri), *P. inui *(13 strains: Leaf monkey 1, Hawking, Cinui, Perak (IM), N34 (IN), Leucophyrus (IC), Perlis (ID), A. hackeri (IF), Leaf Monkey II (IF), Taiwan II (IB), Celebes, Mulligan, and Celebes II) and *P. cynomolgi *(ten strains: T746, T824, B, Cambodian, Ceylonensis, Gombak, Langur, Pt 2, Smithsonian and RO). Most of the parasites were obtained from the American Type Culture Collection (ATCC), unless described previously [16]. Toque monkeys were experimentally infected with *P. cynomolgi *T746 and T828, and a Japanese macaque was infected with *P. coatneyi*, and their bloods collected. These experimental infections were approved by Ethical Review Committee, Faculty of Medicine, University of Colombo, Sri Lanka (No. EC/00/69) and Animal Experiment Committee, Dokkyo University Medical School, Japan (No. 0397). Other sequences retrieved from GenBank are given in Additional file 1, which also lists the parasites' natural hosts.

### DNA sequencing

Parasite genomic DNA was extracted from infected monkey blood using QIAamp DNA Blood Mini Kit (QIAGEN, MD). Nearly complete nucleotide sequences of *msp1 *from Asian monkey parasites were obtained by direct sequencing of PCR products using previously designed PCR primers and sequencing conditions [16]. However, since *P. gonderi msp1 *sequence was not successfully obtained from the above procedures, the uneven PCR method [45] was employed, in which PCR was conducted using a combination of known sequence primer and random primers. Initially, a short conserved region (about 500 bp) in the 5' part of *msp1 *was amplified using Pgo-f92: 5'-TTTTYGTTACCAAATGTCAATGTGAA-3' and Pgo-r2: 5'-ACAACTTTCTTCAGCATGTCCA-3', and sequenced. To extend this short region outwards from both directions, amplifications were done using primers derived from the short region sequence and random primers. PCR fragments were cloned into TOPO TA and XL vector (Invitrogen, Carlsbad, CA) prior to sequencing. Using newly identified sequenced regions as a scaffold for further extensions, the uneven PCR was repeated until the whole *P. gonderi msp1 *coding region was obtained. Finally, the following primers were designed to amplify the full length *msp1*, Pgo-f19: 5'-TGCTACATCTTAAAACATTCGAATAT-3' and Pgo-r17: 5'-TTAAAGCTCACTGCATAGCAGAATA-3'. Cycling conditions were 94°C for 1 min, 40 cycles at 94°C for 20 sec, 58°C for 30 sec, and 72°C for 6 min, and a final extension at 72°C for 10 min. Sequences were obtained using an automated sequencer 3100 Genetic analyzer (Applied Biosystems, CA), and reads were verified at both directions with at least two independent amplifications.

The 6-kb *Plasmodium *mitochondrial genome was amplified using the following primers to cover the complete genome, PvmtF5488: 5'-GGTATAATTCCATTATCTCATCCAGA-3', PvmtR3088: 5'-CAACATAACATTTTTTAGTCCCATGCT-3', PvmtF2959: 5'-TACTAAGATAAAGAACTCCAGGCGT-3' and PvmtR0: 5'-TTAACATAATTATAACCTTACGGTCTGT-3'. PCR conditions and sequencing procedures were the same as those for *msp1*. Sequences obtained in this study have been deposited in DDBJ/EMBL/GenBank with accession numbers AB444049-AB444069 and AB444106-AB444136. We noted an inconsistency of *P. knowlesi *sequences in the present study and the recently published genome report [44]. The sequences of *msp1 *and the mitochondrial genome of the *P. knowlesi *H strain obtained from ATCC did not match the sequence reported in the genome project [44] using the H strain; but those of the ATCC *P. knowlesi *Malayan strain were completely identical to sequences of the genome project. We are uncertain on how to explain this discordance between datasets.

### Sequence alignment

The nucleotide sequences were aligned using Clustal W [46] and alignments were further checked manually. Nucleotide divergence between species and intra-specific nucleotide diversity (π), the average pairwise nucleotide difference per site, were calculated using DnaSP version 4.0 [47]. Average of pairwise amino acid difference was calculated using MEGA version 4 [48].

### Construction of phylogenetic tree

ML phylogenetic trees were constructed using the mitochondrial genome (5818 bp) and *msp1 *(4176 bp) sequences by PAUP v4.0 β10 [49]. In the construction of phylogenetic trees, *P. fieldi *and *P. simiovale *were found to be very closely related to each other (Figures 1a and 1b). Their morphological similarity has also been reported [19]. For these reasons, *P. simiovale *was grouped to the same taxon of *P. fieldi *in this study. Since there are a large number of available *P. vivax *sequences for the mitochondrial genome (n = 282) and *msp1 *(n = 43), two to three distantly related sequences were used for constructing trees. The appropriate nucleotide substitution model was selected by the Modeltest 3.7 [50]. A general time-reversible substitution model where rate heterogeneity among sites was taken into account by a mixture of Gamma-distributed positive rates and a point mass at rate zero (GTR+I+G) was used for both mitochondrial genome and *msp1*. One hundred bootstrap replicates were done for constructing ML trees. Difference between phylogenetic trees of mitochondrial genome and *msp1 *was statistically evaluated using the Shimodaira-Hasegawa test [51] implemented in the PAML version 4 [52].

### Detection of positive selection in ancestral lineages

To assess lineage-specific positive selection on *msp1*, the ratio of kA to kS (= ω), in which kA and kS are the number of nonsynonymous and synonymous changes per nonsynonymous and synonymous sites, respectively, was estimated for branches in a tree using the branch model implemented in PAML version 4. The ω ratio measures the direction and magnitude of selection on amino acid replacements, with values of ω < 1, ω = 1, and ω > 1 indicating purifying selection, neutral evolution, and positive selection, respectively. The heterogeneity of ω among branches was incorporated using the free-ratio model where each branch has its independent ω value. For branches with ω > 1, a LRT was conducted between the free-ratio model and a constrained model with ω of a branch of interest fixed at one.

### Estimation of divergence time of lineages

To date the time frame, in which positive selection is predicted, the divergence time of lineages of interest was inferred from the phylogeny of the mitochondrial genome constructed here. Evolutionary rate constancy was tested for various lineages using Tajima's relative rate test for the molecular clock hypothesis [53]. The mitochondrial genomes of *P. chabaudi*, a rodent malaria species, (GenBank accession number: AB379671) and *P. gonderi *were used as outgroups. When lineages showing rate constancy were identified, the genetic distance between lineages was calculated with the Jukes and Cantor correction. We adopted the divergence time of 10 mya for a common node of *P. gonderi *and Asian monkey malaria parasites, and 6.3 mya for a common node of *P. knowlesi *and other Asian monkey malaria parasites as calibration points [18].

### Detection of positively selected amino acid sites

In order to detect the sites under positive selection, we must be able to distinguish the signatures of selection from recombination. If the sequences have undergone recombination, we can spuriously infer selection within a model that assumes a common phylogeny for all sites [54]. We accomplished this by analyzing datasets independently under population genetic and phylogenetic approaches taking into account the effect of recombination.

The population genetics method employed is the one implemented in the omegaMap software [29]. It is a Bayesian method to infer the posterior distribution of ω (= dN/dS), in which dN and dS are the number of nonsynonymous and synonymous changes per nonsynonymous and synonymous sites, based on a coalescence approach with recombination, where the codon model is an extension of the NY98 model [55] which incorporates a rate for insertion/deletions. In the omegaMap implementation the population recombination rate ρ and the selection parameter ω are allowed to vary along the alignment following independent change-point processes and are sampled through Markov chain Monte Carlo (MCMC). In our analyses we set the prior expected length of blocks for ρ and ω at 30 codons. Two independent MCMC chains were run for 1,000,000 iterations with a burn-in of 10% of the total number of iterations. Upon convergence the two chains were merged to infer ω. Due to constraints in omegaMap, we randomly sampled 25 out of the 43 sequences of *P. vivax*.

The phylogenetic approach assumes that the sequences are free from recombination and consequently all sites follow the same phylogenetic tree. Even when there is a moderate-to-low signal of recombination, we can still infer selection provided that we can detect the recombination breakpoints. In such cases, we may apply the phylogenetic procedure for each non-recombinant segment - defined by the breakpoint locations. HyPhy/Datamonkey [30] is a likelihood-based procedure that can handle potentially recombinant sequences when inferring positively selected sites under the phylogenetic assumption. The recombination breakpoints were first predicted by using the Genetic Algorithm Recombination Detection (GARD) algorithm [56] implemented in HyPhy/Datamonkey. The detection of positive selection was then conducted for each non-recombinant segment. For each segment a neighbor-joining tree [57] was constructed using MEGA 4. To estimate ω (= dN/dS) at each codon, a fixed-effects likelihood method that directly estimates dN and dS at each site was used. This method involves 3 steps: first, the nucleotide substitution model parameters and branch lengths are obtained by ML given the alignment segment and the neighbor-joining tree. Second, values for dN and dS are estimated for each site using the parameters found at the first step and the MG94 codon model [58], with and without the constraint that dN = dS. Finally, an LRT is performed to infer whether dN is significantly different from dS. Owing to computational limitations of GARD, the maximum number of sequences analyzed within the phylogenetic approach was set to 14 per analysis.

## List of abbreviations

MSP-1: Merozoite surface protein-1; *msp1*: merozoite surface protein-1 gene; mya: million years ago; OWMs: Old World monkeys; kDa: kilodaltons; ML: maximum likelihood; LRT: likelihood ratio test; dS: the number of intraspecific synonymous changes per synonymous sites; dN: the number of intraspecific nonsynonymous changes per nonsynonymous sites; kS: the number of interspecific synonymous changes per synonymous sites; kA: the number of interspecific nonsynonymous changes per nonsynonymous sites; MHC: major histocompatibility complex; MCMC: Markov chain Monte Carlo; GARD: Genetic Algorithm Recombination Detection.

## Authors' contributions

KT conceived the study. HS and KT designed the study and drafted the manuscript. HS and HO obtained sequences. HS, NA, LOM, and HK performed the statistical analyses. SP, SH, and SK prepared materials. NP, LOM, HK, TH and KT revised the manuscript. KT obtained funding. All authors read and approved the final manuscript.

## Supplementary Material

Additional file 1 **Malaria parasite species and isolates used in the present study**. Table showing malaria parasite species and isolates as well as accession numbers of sequences obtained in this study and retrieved from the GenBank database. Click here for file

Additional file 2 **Predicted molecular weight of MSP-1 of *P. vivax *and *P. vivax*-related simian malaria parasites**. Table showing variation in molecular weights and amino acid lengths of MSP-1 among *P. vivax *and *P. vivax*-related simian malaria parasites and within several parasite species. Click here for file

Additional file 3 **Amino acid sequence alignment of MSP-1 from *P. vivax *and *P. vivax*-related simian malaria parasite species**. Figure S1 showing amino acid alignment of MSP-1 from *P. gonderi, P. fragile, P. coatneyni, P. knowlesi, P. hylobati, P. inui, P. fieldi, P. vivax*, and *P. cynomolgi*. This figure contains information of sequence regions of four inter-species variable blocks and those used for constructing phylogenetic trees and amino acid sites under positive selection. Click here for file

Additional file 4 **Phylogenetic trees for estimating divergence times among *P. vivax *and *P. vivax*-related simian malaria parasite lineages**. Figure S2 showing a method for phylogeny-based estimation of divergence times among *P. vivax *and *P. vivax*-related simian malaria parasite lineages. Click here for file

Additional file 5 **Positional overlap of positively selected amino acid regions of MSP-1 from *P. vivax*, *P. inui *and *P. cynomolgi***. Figure S3 showing five overlapping positively selected amino acid sequence regions of *P. vivax*, *P. inui *and *P. cynomolgi *that were inferred by the omegaMap. Click here for file

Additional file 6 **Indels and repeats in a variable block (VB4) of *msp1 *from *P. inui***. Figure S4 showing a highly variable sequences due to indels and repetitive sequences in a variable block (VB4) of *P. inui msp1*. Click here for file
