# Supplementary material for: Lineage-specific positive selection at the merozoite surface protein 1 (msp1) locus of Plasmodium vivax and related simian malaria parasites
Source: BMC Evol Biol. 2010 Feb 19;10:52. doi: 10.1186/1471-2148-10-52 (PMC2832629; doi:10.1186/1471-2148-10-52)
Supplement: Additional file 2 — Predicted molecular weight of MSP-1 of P. vivax and P. vivax-related simian malaria parasites. Table showing variation in molecular weights and amino acid lengths of MSP-1 among P. vivax and P. vivax-related simian malaria parasites and within several parasite species. [file 1471-2148-10-52-S2.DOC]

Additional table S2. Predicted molecular weight of MSP-1 of *P. vivax* and *P. vivax*-related simian malaria parasites

| Species | n | Amino acid length | Predicted MW (kDa) |
| --- | --- | --- | --- |
| *P. gonderi* | 1 | 1712 | 194 |
| *P. coatneyi* | 1 | 1929 | 213 |
| *P. fragile* | 2 | 1823 - 1832 | 204 - 205 |
| *P. hylobati* | 1 | 1830 | 207 |
| *P. knowlesi* | 3 | 1821 - 1832 | 202 - 204 |
| *P. inui* | 13 | 1799 - 1918 | 202 - 213 |
| *P. simiovale* | 1 | 1790 | 199 |
| *P. fieldi* | 3 | 1805 - 1862 | 200 -206 |
| *P. cynomolgi* | 10 | 1784 - 1869 | 199 - 207 |
| *P. vivax* | 43 | 1699 - 1769 | 192 - 197 |
