# Supplementary material for: Lineage-specific positive selection at the merozoite surface protein 1 (msp1) locus of Plasmodium vivax and related simian malaria parasites
Source: BMC Evol Biol. 2010 Feb 19;10:52. doi: 10.1186/1471-2148-10-52 (PMC2832629; doi:10.1186/1471-2148-10-52)
Supplement: Additional file 3 — Amino acid sequence alignment of MSP-1 from P. vivax and P. vivax-related simian malaria parasite species. Figure S1 showing amino acid alignment of MSP-1 from P. gonderi, P. fragile, P. coatneyni, P. knowlesi, P. hylobati, P. inui, P. fieldi, P. vivax, and P. cynomolgi. This figure contains information of sequence regions of four inter-species variable blocks and those used for constructing phylogenetic trees and amino acid sites under positive selection. [file 1471-2148-10-52-S3.PDF]

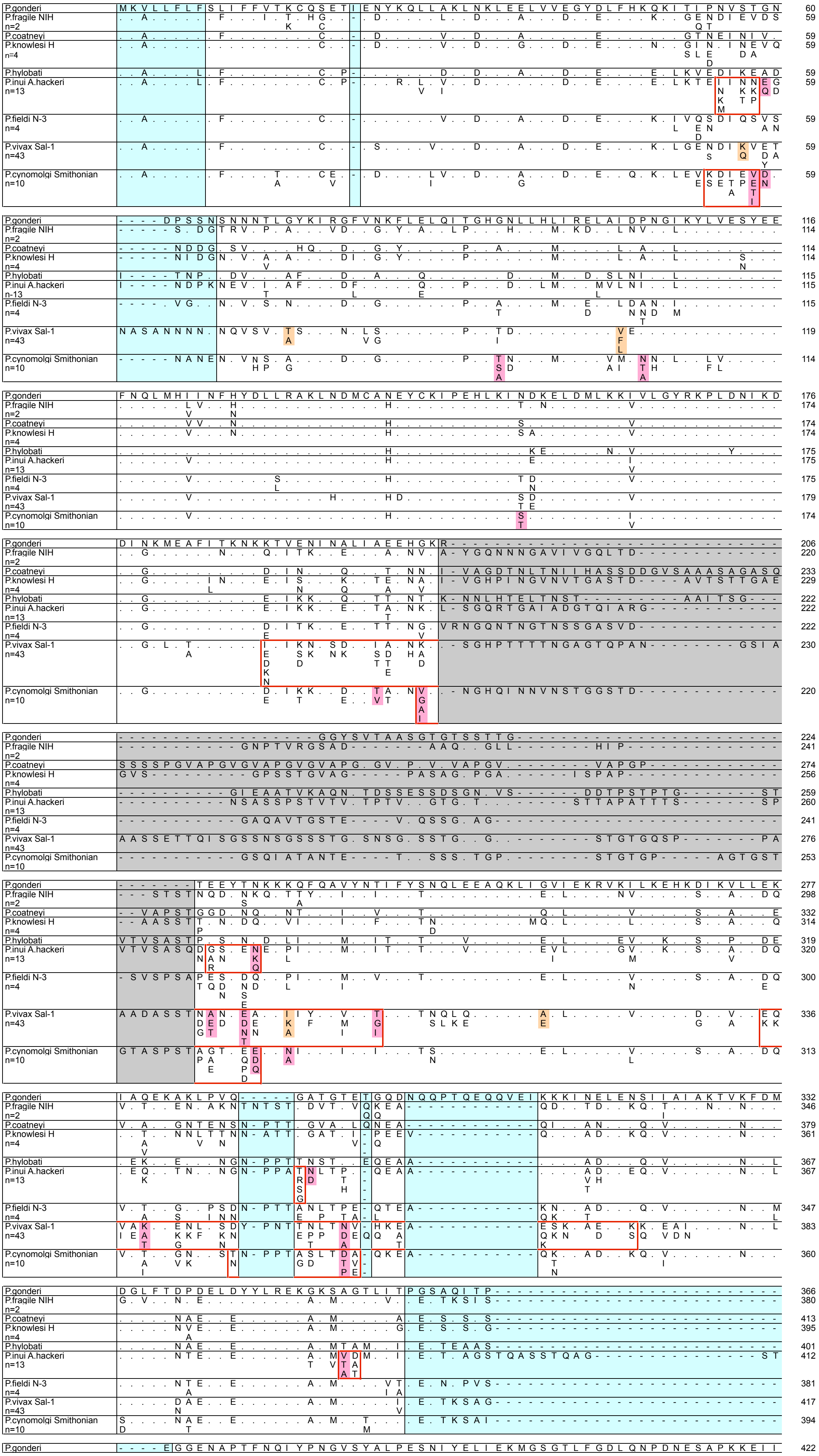
P.gonderi
P.fragile NIH
n=2
P.coatneyi
P.knowlesi H
n=4
P.hylobati
P.inui A.hackeri
n=13
P.fieldi N-3
n=4
P.vivax Sal-1
n=43
P.cynomolgi Smithonian
n=10

```
P.fragile NIH                  - - - - A|P . K T V . . M K E T . . H . I T . . . . . . T . L . . . . N I E . Q E S . . . . . . . . D G Q Q . N G . .      436
n=2
P.coatneyi                     - - - - T|P Q K A V . . L K . T . . H . I . . P . . . S . F . . . G . I . . E E S . . . . . . H D G K Q . S . G . V      469
P.knowlesi H                   - - - - T|P . K A V . . L K E T . . Y . I T . . . . R T . . . . . . . . F . . E E S . . . . . . . . G R Q . N . G . .      451
n=4
P.hylobati                     - - - - A|P K K E V . S M K D S . . H . I T . G . G . . S . F . . . . . L . . E E T . . . F E . . . D A E L . . . . . .      457
P.inui A.hackeri               Q E G S T|P T K T V . S M K E S . . H . I T . G . A . . T . F . . . . . I . . E E T . . . . E . . . D G K L . Q . . . .      472
n=13                                                                 L           L   Y           T     D   S                       E     K
P.fieldi N-3                   - - - - T|T E K A V . . M K E T . . H . I A . . . . . . S . F . . . G . I . . D E S . . . . . . . . D G K Q . . . G . T      437
n=4                                      P     T                         T             N   I               E   T                                 E
                                         S
P.vivax Sal-1                  - - - - T|P . K T V . . L K E T . . H . I . . . . A . N S . . . . . . . I . . D E T . . . . . . . . D G K Q . . . G I L      473
n=43                                     T   N             Q                                                                                     E T I
P.cynomolgi Smithonian         - - - - T|T E K T V . . M K E T . . H . I T . . . . . . T . . F . . . Q . N A . Q E T . . . . . . . . D G K Q . K . G . .      450
n=10                                     P                                             N                   I S   E V S                           N
                                                                                                             G

P.gonderi                      T D E N E R K D L L D K I A N K I K L E E E K L P K L K E E Y E K K L K E Y N E K V K E F L P L L E K F Y E A R L E N T      482
P.fragile NIH                  I N A G T . . L . . . . . M . . . Q E . . . . . . . E . Q Q . . D Q . M E V . K Q . L Q A . . . S . N H . . . . . . N . .      496
n=2                                                                                A                     K L                             Y             H
P.coatneyi                     I S . T K . . L . . . N . T . . . M . . . . . . . . . . . . . . D A . M . L . . . . . E A . . . T . N H . . . . K . D . .      529
P.knowlesi H                   I N . T K . . T . V . . . M S . . . . . . . . L . N . . K E . D E . M K L . K Q . . Q D . . . T . T Y . . . G K . D . .      511
n=4                                                                      F   K       V           E Q     E                     K
P.hylobati                     G S . E K . . . . M . . . M . . . . M . . G . . D N . . K . . . D E . V N N . . . . . S Q . Q . T H K . . . . . . . N . .      517
P.inui A.hackeri               V S . D K . . E . M . . . M N . . . M . . . . . D S . . K K . . D Q . . K . E . . . . D . Q Q T H K E . . . . . . . N . .      532
n=13                                                         S                           I                                       A
                                                                                                                                 I
P.fieldi N-3                   I S . T K . . E . Q D K . T . . . . I . . D . . . D . . K . . D E . F . V . E Q . . N D . K . I . N H . . . . . . . D . .      497
n=4                                  I           I Q                             G           E         E     K       E
                                     S           V                               N
P.vivax Sal-1                  I N . T K . . E . . E . . M . . . . I . . D . . . D . . K . . D E . Y K V . E A . . N E . K . A F N H . . . . . . . D . .      533
n=43                           A     I                                           N           E     F T       Q       K D   Q   E     Y
                                                                                                             E
P.cynomolgi Smithonian         I S . H K . . E . L . . . I . . . . L . . D . . . D . . K . . D E . I . A . E K . . E E . Q . T . H H . . . G . . . . .      510
n=10                             N   I       A   Q                 I             N                     V     Q       K D       I             A
                                     V

P.gonderi                      M E G N K F D E F K N K R S E Y M N K K N D L E K C P Y E Q I T N L I N K L K K Q L T Y L E D Y I L R K E I A N D E I N      542
P.fragile NIH                  L V A E . . . . . . T N . E A . L K E . . E . . . . T . . . N I . . . . . . . . . . V . . . . . . T . . D . . D . . . .      556
n=2                                                                                                                L
P.coatneyi                     F V . D . . . K . . T N . E A . . E M . K E . . . . T . . E K . . . . . . . . . . . . . . . . Q . . N . . . D . T D . . . .      589
P.knowlesi H                   L V . T . . . . . . T . . E A . . K E . E E . . . . T . . . S I . . . . . . . . . . . . . . . V . . T . K . D V T E . . . .      571
n=4                                  S             N     A E
                                                   S
P.hylobati                     I D K Q . . . D . . A . . D . . . K . . G E . . E . T . . . E A D . . . . . . . . . . . . . . . . . T . K . D . . D . . . .      577
P.inui A.hackeri               L V G D . . . A N . . T . . D A . . T E . I E . . . . T Y Q Q E A D . . K . . . . . Q . . . . . . . T . . . D . A N . . . .      592
n=13                               E         P                           K           S H L     N                 H                 N       V D
                                             L                                                 A
P.fieldi N-3                   L V E T . . D A . . T H . E A . . K E . . E . . . . T . . . N T . M . . . . . . . . . . . . . . . S . . . D V S D . . . .      557
n=4                                A A     G E     A             Q                           I   L                                         A N
P.vivax Sal-1                  L V G N . . D D . . K . . E A . . E E . K K . . S . S . . . N S . . . . . . . . . . . . . . . . . V . . . D . . D D . . K      593
n=43                               E E     G E     T       E     K                           T                                               N
                                                                                             A
P.cynomolgi Smithonian         L D . D . . . Q . . A . . E . . . K E . K E . . . . T . . . N A . L . . . . . . . . . . . . . . . N . . . D V . N . . . .      570
n=10                             V                 T                                         I   M                               S       I   D

P.gonderi                      H F S A M E W E L K K E I Y K L E K E I K K N E S K I T I D N Q F E L S D V V E L Q V Q K V L I M K K I E A L K N V K N      602
P.fragile NIH                  Y Y . D . . . K I . N . . . E . A R . . Q . . . I . L I V E . K . D F . G . L . . . . . . . . M L . . . . . . . . . A Q .      616
n=2                              F                             D                       D
P.coatneyi                     Y . . H . . . K . . S . . F E . A . . . R . . . . . L I T E . K Y D F . G . . . . . . . . . . M I . . . . . . . . . . Q .      649
P.knowlesi H                   Y . . D L . . K . . N . . . E . A . . V R . . . N . L I M E . K . D F . G . L . . . I H . . . M I . . . G . . . . . . Q .      631
n=4
P.hylobati                     S . . . . . . K . . S R . . E . S . . V R . . . N . M V L E D K . D F . G . . . . . . . . . . I . . . . . . . . . . I Q .      637
P.inui A.hackeri               S . S . . . . K . . S R . . E . S . . V R . . . N . L V L E . K . D F . G . . . . . . . . . . I . . . . . . . . . . I Q .      652
n=13                               R
P.fieldi N-3                   Y F . D . . . K . . N E . . G . A K . . Q . . . N K L . V E . K . D F . G . . . . . . . . . . I . . . . . . . . . . I Q .      617
n=4                              L   H             D         Q             S E                 I
                                     G
P.vivax Sal-1                  . . . F . . . K . . S . . . D . A Q . . R . . . N . L T I E . K . D F . G . . . . . . . . . . I . . . . . . . . . . . Q .      653
n=43                                                                             I V
P.cynomolgi Smithonian         Y F . T . . . K . . N . . . E . S K . . R . . . S . L . V E K K . D F . G V . . . . . . . . . N . . . . . . . . . V Q .      630
n=10                             Y   H                         M           N       A   N             I               I                   I

P.gonderi                      L L K N A K V K D D L Y I P K V Y K T V Q K P E P Y Y L I V L K R E V D K L K D F I P K I H K M I E A E K A K Q|- - - -      658
P.fragile NIH                  . . . . . . . . N G . . . . . . . . S G D . . . . . . . . . . . . K . I . . . . . . . . . . . E T . . T T . . . . S|- - V T      674
n=2                                            D
P.coatneyi                     . . . . . . L . D G . . . . . . . . G E . . . . . . . . . . . . . K . I . . . . . . . . . . . E T . . A T . . . N S|- - A T      707
P.knowlesi H                   . . . . . . L . . K . . . . . . . . G . . . . . . . . . . . . . . K . I . . . . . . . . . . . E T . . A T . . . . A|- - P T      689
n=4
P.hylobati                     H . . . . . . . . . . . . . . . . . G E . . . . H . . . . . . . . K . I . . . . . . . . . . . E A . . T T . . . N L|- - A K      695
P.inui A.hackeri               H . . N . . V . . D L . V . . . . . G E . . . . H . . . . . . . . K . I . . . . . . . . . . . E A . . T T . . . N L|- - A K      710
n=13                                 K     L     N F               V
P.fieldi N-3                   L . . . . . . . . N . . . . . . . . G E K . . . . . . . . . . . . K . I . . . . . . . . . . . E T . . T T . . . . T|- - P T      675
n=4                            H                                       T
P.vivax Sal-1                  . . . . . . . . . . . . I . . . . . S E . . . . . . . . M . . . . . I . . . . . . . . . . . E S . . A T . . N . P|T V A A      713
n=43                                                   V           G                                                       Q A       T       A
P.cynomolgi Smithonian         . . . . . . . . . D . . . . . . . . G E . . . . . . . . . . . . . K . I . . . . . . . . . . . E T . . T T . . . . S|- - G T      688
n=10                                             G                 D G                                                                         T

P.gonderi                      -|T A P A T G T I V A Q D E R|S T S S T P S S A A A P T E T S - - - - - - - - - - - - - - - - - - - - - - - - - - - - -      688
P.fragile NIH                  A|P V V I K . Q S L R G A S E|T K E T A T T . P V G D A V G A A G Q - - - - - - - - - - - - - - - - - - - - - - - Q G T      711
n=2
P.coatneyi                     E|P . N V S . Q S L R G A S E|T V T G A V T . T V G T . G S Q T A S E T S E Q S G N Q S Q G P P P Q G G N K P Q G P P P      767
P.knowlesi H                   E|P V K V R A Q S L R G A S E|T A P . A . P A Q P . A . T E T Q S V P S E A S A K P T P T A - - - - - - - - T E S G S G      741
n=4
P.hylobati                     E|A . D . . T Q S . K G E S E|T V T V A G G . T . . T . T A N A S E N A A G T - - - - - - - - - - - - - - - - - - - - -      734
P.inui A.hackeri               E|A A A T Q G Q S L R G E S E|T . . P A . P A P P T V P A A P T V P A A P T V P A - - - - - - - - - - - - - - - - A P A      754
n=13                              V S S T E
                                  T     P
P.fieldi N-3                   S|A V Q I R . Q S L R G A S E|T G A T G V T . D V G T S A A . A S - - - - - - - - - - - - - - - - - - - - - - - - - - -      708
n=4                                 P       E             D
P.vivax Sal-1                  A|D I V A K . Q S L R G A S E|T G T T G N T - - - - - - - - - - - - - - - - - - - - - - - - - - - - - - - - - - - - -      735
n=43                            A P   T S     L       S
P.cynomolgi Smithonian         E|A V Q I K . Q S L R G A S E|T G V T P T P P . P . . A L S P A V G A T - - - - - - - - - - - - - - - - - - - - - - - -      724
n=10                                  V

P.gonderi                      - M Q Q T S S D G S Q Q P Q - - - - - - - - - - - - - - - - - - - - - - - - - - - - - - - - - - - - - - - - - - - - - -      701
P.fragile NIH                  G Q T A . E G S A G . G E . A - - - - - - - - - - - - - - - - - - - - - - - - - - - - - - - - - - - - - - - - - - - - -      726
n=2
P.coatneyi                     P P A S V . P Q A E S S T P Q - - - - - - - - - - - - - - - - - - - - - - - - - - - - - - - - - - - - - - - - - - - - -      782
P.knowlesi H                   S T T S A T T A V Q . P . . P - - - - - - - - - - - - - - - - - - - - - - - - - - - - - - - - - - - - - - - - - - - - -      756
n=4
P.hylobati                     - - P A V N T . A T P A E N - - - - - - - - - - - - - - - - - - - - - - - - - - - - - - - - - - - - - - - - - - - - - -      746
P.inui A.hackeri               A P A P L A . H T T T S D T - - - - - - - - - - - - - - - - - - - - - - - - - - - - - - - - - - - T T S N T A T S N T      778
n=13
P.fieldi N-3                   - Q . S Q Q T Q V E T A . A A - - - - - - - - - - - - - - - - - - - - - - - - - - - - - - - - - - - - - - - - - - - - -      722
n=4
P.vivax Sal-1                  - V N A Q T A V V Q P . H . V - - - - - - - - - - - - - - - - - - - - - - - - - - - - - - - - - - - - - - - - - - - - -      749
n=43
P.cynomolgi Smithonian         - T . T A Q G A A T P G A A - - - - - - - - - - - - - - - - - - - - - - - - - - - - - - - - - - - - - - - - - - - - - -      737
n=10

P.gonderi                      - - - - - - - - - - - - - - - - - - - - - - - - - - - - - - - - - A H V E S T I Q S T P V Q G S Q P N - - - - - - - - - -      719
P.fragile NIH                  - - - - - - - - - - - - - - - - - - - - - - - - - - - - - - - V T E T E . T P T P - . A Q T E K S E S V V E K S T H - - -      752
n=2
P.coatneyi                     - - - - - - - - - - - - - - - - - - - - - - - - - - - - - - - T A S L P A V P G E A P A T T Q V . A - - - - - - - - - - -      801
P.knowlesi H                   - - - - - - - - - - - - - - - - - - - - - - - - - - - - - - - A A G T T S T . A P A E G G A Q E V T Q - - - - - - - - - -      776
n=4
P.hylobati                     - - - - - - - - - - - - - - - - - - - - - - - - - - - - - - A D A T P A . N A D A T P A E N A D A T P - - - - - - - - - -      767
P.inui A.hackeri               A T E I S T S D T T T S N T A T S N T A T E I S T P A T T T S N T A T . I S T P A . T T P A T T T S - - - - - - - - - -      828
n=13
P.fieldi N-3                   - - - - - - - - - - - - - - - - - - - - - - - - - - - - - - - P A . P . P Q V . T A Q . T S Q . P T - - - - - - - - - - -      741
n=4
P.vivax Sal-1                  - - - - - - - - - - - - - - - - - - - - - - - - - - - - - - - V N . V T V Q P G T T G H Q A Q G G E - - - - - - - - - - -      768
n=43
P.cynomolgi Smithonian         - - - - - - - - - - - - - - - - - - - - - - - - - - - - - - - - - - - A A T . T T T G G G E - - - - - - - - - - - - - - -      748
n=10

P.gonderi                      - - - - - - S V S N P G A E E G T S A P E T N N E A T E V Q E Q A S - - - - - - - - - - - - - - - - - - - - - - - - - - -      747
P.fragile NIH                  - - E T T T T G G P T V E . S A I T D K Q P A E S E A T H E T P P A - - - - - - - - - - - - - - - - - - - - A I G E G A Q      791
n=2
P.coatneyi                     - - - - Q P A P P E E P P V A Q A T V . V P E T . E Q S A P T E P A - - - - - - - - - - - - - - - - - - - - G G E E A G A      838
P.knowlesi H                   - - - - - S E E T A T P P G P E V P . T P - - - - V P G E T . . Q Q - - - - - - - - - - - - - - - - - - - - E Q Q - - - -      804
n=4
P.hylobati                     - - - - K A M S V E D L K A M T K E M . . E T L R . I T T P T T . E - - - - - - - - - - - - - - - - - - - - N P E T N T E      804
P.inui A.hackeri               - - - - - A T T T S A T T T S A . T T S A . T T S . . T T S A T T T S A T - - - - - - - - - - - - - - - - - T S A T T T P      867
n=13
P.fieldi N-3                   - - - - Q A A P A P T P . . - - S P S . A . L P S E . - A T T A P T - - - - - - - - - - - - - - - - - - - - E T S T T G T      775
n=4
P.vivax Sal-1                  - - - - - - - - - - - - - - - - - - . E T Q T . S V Q A A . V . Q T - - - - - - - - - - - - - - - - - - - - P A G A G G Q      791
n=43
P.cynomolgi Smithonian         - - - - - T Q A A G V A . G V E A P E G A E S Q P . G A A P Q P . G - - - - - - - - - - - - - - - - - - - - A A P Q P A G      784
n=10

P.gonderi                      - - - - - - - - - - - - - - - - - - - - - - - - - - - - - - - - - - - - - - - - - E E S S S - - - - - - - - - - - - - -      752
P.fragile NIH                  T T S S P E E P T E A E A - - - - - - - - - - - - - - - - - - - - E V P A P G K S A T T A E E P A - - - - - - - - - - - -      820
n=2
P.coatneyi                     E T A A P G G E A T - - - - - - - - - - - - - - - - - - - - - - - Q E Q E A A A E A P Q E S Q Q P P T E P A G      870
```

P.knowlesi H<br>n=4	- - - - - - - Q A A E E G - S Q N E N A Q Q E G - - - - - - - - - - - -	820
P.hylobati	T D T A A T G V T - Q . . E - - - - - - - - - - - - -	817
P.inui A.hackeri<br>n=13	A T T T P A E T T P A E T T P A E T T P A E T T P A T T T E I S I P T T T P A A A T P A T T T - - - - - - - - - - - - -	914
P.fieldi N-3<br>n=4	E P A V P E G E S S - S E E E A T E G G - - - - - - - - - - - - -	794
P.vivax Sal-1<br>n=43	V A S T - Q T I S Q A P A P T - - - - - - - - - - - -	805
P.cynomolgi Smithonian<br>n=10	S A P Q P A G A S - P P V A P E E P - - - - - - - - - - - -	801

P.gonderi	- S E N P E N A S P A A P P A - - - - - - - - - - - Q A A P A M T K L E Y L E K	780
P.fragile NIH<br>n=2	- - - - - - - - - - - - - - - - - - - Q K T . A . A D . A A . T T . . P A P V - - - - - - - P . V . T . S	854
P.coatneyi	G A A A P A E T E D V T T Q E A P A Q P A E P T . A A T A . V . T A P A T T T - - - - - - - P . V . P . S . . Q	923
P.knowlesi H<br>n=4	- - - - - - - - - - - - - - - G S A T P T E T T . V E T T T . V T . P A T T A - - - - - - - P . . . . S	858
P.hylobati	- V D T I . T T T P A - - - - - - - - - - - - - - - - P . E A S . S . V	841
P.inui A.hackeri<br>n=13	- - - - - - - - - - - - - - - - - - - E I S I . T T T P T P . A A L S P A L S - - - - - - - P . . S . S . V	948
P.fieldi N-3<br>n=4	- - - - - - - - - - - - - - - - - - E T P P A V . G A . T . G . T . G A T P A A - - - - - - P . . . S . S	830
P.vivax Sal-1<br>n=43	- - - - - - - - - - - - - - - - - - - Q A . P E . A P . A . P S T . . A A V - - - - - - - - A P . . T . S . . . . . E .<br>A Q	838
P.cynomolgi Smithonian<br>n=10	- - - - - - - - - - - A P E E P V G - - - P V T . V T T P Q P T S Q P A A P T A - - - - - - P . . . T . S	841

P.gonderi	L L E F L K S A Y R C H K H I F V N R S T M G K E A L T K Y E L N T L E S R I I N V S P C D E L D L L F N V Q N N L P A	840
P.fragile NIH<br>n=2	. . D . . . . . A . . . . . . L T N T . . N P . L . K Q . K . T P D . Q N R . . H N S . . . . I . . . . . .<br>T K I Q	914
P.coatneyi	. . . . S . A . . . . . . L T N . . . S P Q L . E Q . K . T A D . E T Q . K Q . T . . Q . . . . . . I	983
P.knowlesi H<br>n=4	. . . . S . A . . . . . . L T N . . . N P . L . K Q . A . T . D . E K K . K E . A . . . . S	918
P.hylobati	. . N . . . . . A . . . . T N . . . K E . L . K E . . . T . D . E E K . K Q . . . . N . . . I	901
P.inui A.hackeri<br>n=13	. . N . . . . . A . . . . T N . . . K . . L . D Q . K . T T D . E N K . K E . K . . K . . . I . . . V . . . . . A<br>E A T N L S	1008
P.fieldi N-3<br>n=4	. . D . . . S . A . . . . . . I T N . . . N . D L . S Q . . . T A E . K N K . K E . T . . Q . . . . . . V . . . . . .<br>V M D S N K	890
P.vivax Sal-1<br>n=43	. . D . . . . . A . . . . T N . . . K . E L . D Q . K . . A D . Q N K . K E T K . . . .<br>D K K E E . K T N Q N	898
P.cynomolgi Smithonian<br>n=10	. . D . . . S . A . . . . T N . . . N . . L . K K . E . T D D . K N K . K Q . T . . E . . . .<br>E Q A A E Q<br>T	901

P.gonderi	M Y S I Y E S M S N D L Q N L Y I E L Y E K E M I Y S I Y K N K K D D K K I K A L L E T P A S N T P P T T T A A D G T	900
P.fragile NIH<br>n=2	. . . . . D T . I . . . . D . . Q . . . . . N . . . R . D T . T - . . . . F . . A S N . . A A A A V A P . P A S<br>N	973
P.coatneyi	. . . M . D T . T . . . . Q . . . L . N . . . D . D T . S - . . . . F . K P S T . . A A E A A A . P A Q S	1042
P.knowlesi H<br>n=4	. . . . . D T . I . . . . Q . . . V . N . . . . . D T . T - . . . . F . . . L K . . A A A A A V T P A V V	977
P.hylobati	. . . L . . T . G D . . . . N . . . . . D . . T - R L . V . . . . - - - - - - - - - - - - - -	945
P.inui A.hackeri<br>n=13	. . . . . D T . . D . . . . N . . . . . E . . T - R L . . . . . . - - - - - - - - - - - - - -<br>D	1052
P.fieldi N-3<br>n=4	. . . . . D T . I . . . . Q . . . V . N . . . . . D T D T - . . . . F . E . L T . . A A A A P . . A V A<br>S N N D	949
P.vivax Sal-1<br>n=43	. . . . . D . . . N E . . . . Q . . . V . N . . . . . D T . K - . I . . F . . . L K . K A A A P - - - - - - -<br>S N M L<br>T	950
P.cynomolgi Smithonian<br>n=10	. . . . . D T . I . . . . E . . Q . . . L . N . . . . . D T . A - . . . . F . . . L T . . . - - - A A A . S A A A<br>D V T	957

P.gonderi	S T - - - - - - N T E V S S P P V A P - - - - - - - - - - S V P A S P D T A V A E T L N T A A S T T P Q D A Q T S D S S	944
P.fragile NIH<br>n=2	A A - - - - - - P P T - T P S T T S - - - - - - - - - - - - P T T . . A V . P . Q S A V N V T D . V V T N P V A T T T T	1014
P.coatneyi	A E - - - - - - . . N D Q V V . N P E P T - - - - T T T T T T T T T . T T T V D Q . N T . . E . V T N E V S T A T P	1091
P.knowlesi H<br>n=4	P - - - - - - - - A P - - A E . . V T P P - - A A P A V V T P T E P A A A P T T T N Q S . T P . G . T T N . V S P T T A	1025
P.hylobati	- T N P V . T T T T	954
P.inui A.hackeri<br>n=13	- T N P V . T G G A	1061
P.fieldi N-3<br>n=4	P V - - - - - - S A P - A - - - - - - - - - - - - - - - - - - - S A S A A . P V Q S A A N L N . Q V V T N P V . T T T T	983
P.vivax Sal-1<br>n=43	- . Q S A A K P S G Q A G T T P V . T T A P	971
P.cynomolgi Smithonian<br>n=10	A A - - - - - - P . A - A V V . - - - - - - - - - - - - - - - - - - A T A V T P . Q P A E D S N E E V V T . L A N T T T -	992

P.gonderi	- A T P G G T S G V -	953
P.fragile NIH<br>n=2	T T T P T T T T T T T T - - - - - - - - V . . T Q D S -	1034
P.coatneyi	- V A . . T Q G P A - S A D T Q A	1106
P.knowlesi H<br>n=4	- V . . A Q D T -	1033
P.hylobati	- V S Q D N S T S -	962
P.inui A.hackeri<br>n=13	- V S Q . N S T S -	1069
P.fieldi N-3<br>n=4	- V S Q N N P T S G T V T N T V S T T Q T V A P G T Q A P A A T S - - - - - - - - T	1016
P.vivax Sal-1<br>n=43	- V . T T T V T P -	979
P.cynomolgi Smithonian<br>n=10	- T A . . N P E S -	1000

P.gonderi	- - - - - - - - - - - G A V D T A T T T T E - - - - - - - - - - - - T Q N E Q K V T A S S S D D V T Q E N A I N D K E L Y	991
P.fragile NIH<br>n=2	- - - - - - - - - - - D . T N . Q . . L - - - - - - - - - - - - - - Q T E Q N V S V I . A . N E . E P K V T I V Q M E K P .<br>R	1070
P.coatneyi	S - - - - - - - - - - P V A . . Q A S P V T E T Q D T T Q A E Q N Q T E Q D Q T V Q A . . E E E S E T . I V Q M D K I .	1156
P.knowlesi H<br>n=4	- - - - - - - - - - - A Q T T . E N A . E - - - - - - - - - - - - - - E E T S V S V Q A . . E E E P E T . I V N V E K I .<br>G G T H	1070
P.hylobati	- - - - - - - - - - - V V T . R E A A P - - - - - - - - - - - - - - Q - A G E Q I V K A N C . . N P E D L L M Q M E M I .	997
P.inui A.hackeri<br>n=13	- - - - - - - - - - - V V T N . Q . . P - - - - - - - - - - - - - - H E E G S H T V K A N C . . K P E D L L M Q M E M I .<br>A Q S<br>T<br>P<br>N	1105
P.fieldi N-3<br>n=4	Q P S V V T N T P A S V E T N A Q . . P - - - - - - - - - - - - - - P G E G S L T V Q A N . E E E A E A . N V K L E D I .<br>E N T A G S D P D L D<br>L<br>Q	1063
P.vivax Sal-1<br>n=43	- - - - - - S P Q T S V V T S . P P . P - - - - - - - - - - - - - - Q A E E N R R V G G N . E E K P E A D T A Q V E K F .<br>V S Q P A E E S V V L I<br>A<br>T	1020
P.cynomolgi Smithonian<br>n=10	- - - - - - - - - - - A V T N . Q . . P - - - - - - - - - - - - - - Q A E G D L P V G G . . E E E S E V K I E E L K N I .<br>E Q A S A P A F E<br>V L E<br>S<br>R	1036

P.gonderi	E K H L S E V G K Y D Y Y F K K F V E S K K D E I N K M D D T N W N K L G E E I E L L K K K L Q V S L D H F D K Y K L K	1051
P.fragile NIH<br>n=2	D R H . . Q M D T . N D . . A . . L . . Q . E N . . N . N K E Q . E T . . A . . . Q . . E . I . . . . . . Y G . . . . .<br>Y	1130
P.coatneyi	. N . . . Q M D Q . . E . . M . . L . . Q . E . . . . N . T N E K . E A . . T . . . A . . . V . . . . . . Y G	1216
P.knowlesi H<br>n=4	. . . . . Q M D . . N D . . I . . L . . Q . E K . T S . T E E Q A . A . . A . . . A . . . V . . . . . . Y G . . . . .<br>I T I	1130
P.hylobati	. . . I . . . M D . . N E N . N . . L . . N . E K . T T . T E Q E A K A . . A . . . Q . . . I . . . . . . Y G	1057
P.inui A.hackeri<br>n=13	. . . I . . . M D . . N E N . N . . L E . . . Q K . T T . K D E E . K A . . T . . . Q . . . I Q . . . . . Y A . . . . .<br>A I E E K H G	1165
P.fieldi N-3<br>n=4	. . . . . Q I D . . N D H . . . . L . . Q . E R . T G . . P A Q . K V . . V . . . E . . . I Q V . L . . . G . . . . .<br>Y E S N A I G E I V<br>L	1123
P.vivax Sal-1<br>n=43	. . . . . Q I D . . N D . . Q . . L . . Q . D . . . T . . . E . K . K A . . A . . . E . . . . Y G . . . . .<br>K K E I D N K	1080
P.cynomolgi Smithonian<br>n=10	. . H . . Q I D N . N D . . . . . L E . . Q . E N . T K . D . T Q . K A . . V . . . E L . . . I . . . . D . Y E . . . . .<br>N K K Q Q S A T K V N	1096

P.gonderi	L E R L L T K K N K I S S S K G N V K N L A K L K A K L E R R Q N L L N N P T N V L K N Y T I F F N K K R E S E K K T I	1111
P.fragile NIH<br>n=2	. . . F M K . . K . . . . . . N . . E H I . K . T S . . N . . . K . K K F . . . . . S S . . . . . V . . . . . K . A . R . E V<br>N	1190
P.coatneyi	. . . F . E . . . . N . . E H I . K . T S . . N . . . . S . S . . . . A V . . . . A . . . E V	1276
P.knowlesi H<br>n=4	. . . F . E . . . . N . . E H I . K . T S . . N . . . . K L . F . . . . . S . . . . I V . . . . . K . A . . . E V<br>I	1190
P.hylobati	. . . F . K . . E . V T D . . R Q I . R . T I . . N . . . . . . R . . . I . . S . . . . F S V . . . . M E V	1117
P.inui A.hackeri<br>n=13	. . . F . K . . K K V T H . . G Q I . G . T I . . N . . . . Q Q . . . I . . S . . . . F . V . . . . E V<br>N E N R E R R K<br>T	1225
P.fieldi N-3<br>n=4	. . . F L K . . . . N . . E Q I . K . T S . . N . . . . S . . . . . A V . . . . A . . . E V<br>M S	1183
P.vivax Sal-1<br>n=43	. . . L . K . . . . N . . D Q I . K . T S . . N . . . . S . . . . . A . . . . T . . . E V<br>F	1140
P.cynomolgi Smithonian<br>n=10	. D . F M K . . . . N . . E Q I . K . T S . . N . Q . . . . S . . . . . V . . . . A . . . E V<br>E T E	1156

P.gonderi	E N T L K N T D I L L K Y Y K A R A K Y Y I G E S F P L K N I S K E S I Q K E E N F L N L E K F R V L S R M E S R L G K	1171
P.fragile NIH<br>n=2	. . . . E L . . . . Q . . . . P . . . T L . E . F L . . . D . Y . . . . G . . . N<br>S	1250
P.coatneyi	. . . . E . . . . P . . . T L . E . L . . . D . Y . . . . . K . . . . G . . . N	1336

Species	Sequence	Position
P.knowlesi H	. . . . E . . . . P . . . . T L . E . . L . . . D . Y . . . . G . . . N	1250
n=4		
P.hylobati	. . . . E . . . N . . . . L . . . . . S . P . . . . T . . E . . L . . . D . Y . . . K . . . . G . . . N	1177
P.inui A.hackeri	. . . . E . . . N . . . . L . . . . M S . P . . . . T . . E . . L . . . D . Y . . . K . . K . . . . . . G . . . D	1285
n=13	K I R	
	D	
P.fieldi N-3	A . . . . . . E . . . . P . . . . T L . E . . L . . . D . Y . . . . M . G . . . N	1243
n=4	M L	
P.vivax Sal-1	. . . . E . . . . I . . P . . . . T L . E . . M . . . D . Y . . . . L . G	1200
n=43	M L	
P.cynomolgi Smithonian	. . . . E . . . . P . . . . T L . E . . M . . . D . Y . . . . R . . . . . . M . G . . . N	1216
n=10	L K I	

Species	Sequence	Position
P.gonderi	N I D L E K E N I S Y L S S G L H H V L T E L K E I I K N K S Y T G S D Y S K N I E E V K K A L E E Y K D L L P K V E T	1231
P.fragile NIH	. . N . . . . T . T . . . . F . . . . S . . R . . . . . H K . . S . A . . . . . Q . . Q . . . . . . A .	1310
n=2	N	
P.coatneyi	. . N . . . . F . . . . . V M S . . K . S . . E H A . . T A A . . E . . Q A . Q E . . . . . A .	1396
P.knowlesi H	. . N . . . . F . . . . K . . . N . H A . . T T A . . E . . Q A . E E . . . . . A .	1310
n=4		
P.hylobati	. . K . . . . F . . . Q . . . T D . K . S . K . H N . . A Q . . . . A . Q E . I . . . A .	1237
P.inui A.hackeri	. . Q . . . . F . . . . D . . K . S . N . H N . . A . K . . D . . R A . Q E . I . M . A .	1345
n=13	K K R E K	
	Q	
P.fieldi N-3	. . . . F . . . . S . . R Y T . N E H A . . T A A . . E . . Q A . Q E . . . . . G .	1303
n=4	G F P S D D V	
P.vivax Sal-1	. . E . . . . H . . . . K N . K . S . N . H T . . . A A . . E . . Q A . Q E . I . . . T T	1260
n=43	L N D K A E K S	
	D	
P.cynomolgi Smithonian	. . . . F . . . . N . . R . S . N . H A . . T A A . . V . . Q A . Q E . . . . . T .	1276
n=10	S S E V	

Species	Sequence	Position
P.gonderi	T - T - - - - - - - - - - - - A S P - - - - - - - - - - - - - A Q - - V T P P A Q V T P P A Q A E A - - - - - - - - - - - - -	1253
P.fragile NIH	K A S P S L P - - - - A A P . A . - - - - - - - - - - - - - . . P - . . . . V T P A E E T E E - - - - - - - - - - - - - -	1339
n=2		
P.coatneyi	Q - . A S L P A P P A T P P V A . A T T Q V Q A P - - - P E E P S . A . S T V P V . V P E T - - - - - - - - - - - - - - -	1438
P.knowlesi H	Q - . A S L P - - - - - - P V A . - - - - - - - - - - - - - - P A . V . . V A P E A E . E . - - - - - - - - - - - - - - -	1335
n=4		
P.hylobati	Q E . A R V P V T P A I T Q G P E Q P V A P E A P - - - E E E A G E E T A . E E A A V E A V A G - - - E T T P V T T - -	1289
P.inui A.hackeri	Q E . A R V P V A L A A T Q G P A S A V T P A V A - - - P E A P T A E E A . E E A A V E A . A G - - - - - A P A S G - -	1395
n=13		
P.fieldi N-3	Q V S A S V P A L P A G A G . A . A V P V P V A E - - - G . P A A S A D V P A A P A V P G . G - - - - - - - - - - - - - -	1347
n=4		
P.vivax Sal-1	Q E G A S T T A A T L P V T V P - S . V P G G L P G . G - - - - - - - - - - - - - -	1287
n=43		
P.cynomolgi Smithonian	Q E G A S V P A A G A G - - - - - - - - - V V G T - - - P . A V I A A . . . A G A G - - - - - - - - - - - - - - - - - -	1306
n=10		

Species	Sequence	Position
P.gonderi	- - - - - - - S Q T S E V A V E P V - P Q E G A A E G T S G A A T	1278
P.fragile NIH	- - - - - - S E E S A P T . P P V K Q E A - G P T . E S P V A A E T A	1367
n=2		
P.coatneyi	- - - - - - E T E . E . Q P A . E . S E G - G . . . G G A A A P . . P	1466
P.knowlesi H	- - - - - - G A G . P T S . E P A T A D T - A D T . D T A A P T Q T S	1363
n=4		
P.hylobati	- - - V T D A A T P A . T . T P A E T A T P D A P P A E V P - - - - - - - - - - - - - A Q V P A A G P G S A P E P A . . P	1334
P.inui A.hackeri	- - - E A P A G A A P A . T . P S A T V R A G A T T T T - - - - - - - - - - - - - - - - - - T Q G G V G E A . A T T T T I	1435
n=13		
P.fieldi N-3	- - - - - - A P A A . A D . Q A A S - A G A P E A S A G A S E A	1372
n=4		
P.vivax Sal-1	- - - - - - V P G A A A G L T P . - P P . G - - S V P . T G	1308
n=43		
P.cynomolgi Smithonian	- - - - - - - A G P A A G Q T P G A - A P A P E T A P A A P G T .	1331
n=10		

Species	Sequence	Position
P.gonderi	- P A P I P T S P S T A E S T S T L - - - - - - - - - S	1296
P.fragile NIH	A P A A P A Q P V T P P V T P - - - - - - - - - - - - - - - - - - - A E E . A A G . V Q T . P A A G G E G Q T G Q T T	1407
n=2		
P.coatneyi	G E P E A S L P A P E G E T S E E T - - - - - - - - - - - - A Q A Q . . A Q A Q A Q . P A Q . P A G E T A P L A Q P E A	1514
P.knowlesi H	A A Q P A T A D T A A Q P A T A D T - - - - - - - - - - - - A A Q . . T A D . A A Q P . I A P A A V P E N G E T A E V K	1411
n=4		
P.hylobati	A P E P A P A A A P A A V P T A A P A P E P - - - - - - - A P A A . V A V . E A E V P E V . . T . T Q S G Q G E G L V .	1387
P.inui A.hackeri	T T Q G G V G E A G A T T T T T T T I T T Q - - - - - - - E D E G S T T T T . T T I . T T T . T . - Q G G A G S T S V I	1487
n=13		
P.fieldi N-3	S A G A P A A L P A G A P T T G E T - T G E . T G E . A - - - - - - - - - T V T T	1403
n=4		
P.vivax Sal-1	- - P G A A A G S T E E N - V A A K	1323
n=43		
P.cynomolgi Smithonian	P A P E V A A V A E G - T P G A E G E G L T Q . - - - - - - - - - - - - - - Q	1355
n=10		

Species	Sequence	Position
P.gonderi	G Q D D T E D D E K V I A L P L F - - E E K M D A Y D - - - - - - - - E E Q V T M G E A E E - R Q V E V I V P R D L N E	1345
P.fragile NIH	Q D V Q A Q . Y D E . . . . G N N D D D G D . . E - - - - - - - . D . . . T . . . . N - A . P . . . I . E G I S .	1459
n=2	K	
P.coatneyi	A . V S G D . Y D Q . . . V . . I G N D . E D . E N E E - - - - - - - V N . . . T . . . . N - A . P . . L . . E G I . .	1566
P.knowlesi H	A . E Y G . . Y D . . . T . . . . G N D . D D V E D Q E - - - - - - - . K . I I T . . . . N - A . P . N . . . E G I . .	1463
n=4		
P.hylobati	. . N Y E D . Y D . F R F H . F V N K N D D - - Y V E E E E E N K E D A D . . . T . . . . A E E T P . . L . . E G I . .	1445
P.inui A.hackeri	A . . Y D . . Y D . F R F H . F V Y K N D D E G Y D E E E E E N - K E V D . . . T . . D . N Q E T P . . . . . P G I . .	1546
n=13	G A E K S E	
	L	
P.fieldi N-3	A . . Y A . . Y D . . . . G N N D D D G - - E E - - - - - - E A E Q . I S . . T . N - A E P . I . . . Q G I . .	1454
n=4	A V D H T V P	
P.vivax Sal-1	A . . Y A . . Y D . . . . G N N D D D G E - - - - - - - - - - . D . . . T . . . . S - E A P . I L . . A G I S D	1372
n=43	E	
P.cynomolgi Smithonian	A . . Y A . . Y D . . . A . . . . G N N D D D G D D K E - - - - - - - A D . . . . S - E A P . I . . . Q G I . .	1407
n=10	D K K	

Species	Sequence	Position
P.gonderi	Y E V V Y M K P L A E V Y K T I K K Q L E N H L N A F N N N T M D M L E S R L K K R N Y F L S V L N S D L A P Y E R A S	1405
P.fragile NIH	. . . . . I . . . . G I . N . . . . A . . V A . . . T . I I . . . D . . . . N . . . . E . . F N . F K Y S .	1519
n=2	S	
P.coatneyi	. . . . . I . . . . G M . . . . A . . V A . . . T . I T . . . D . . . . A . . D . . N . F K Y P .	1626
P.knowlesi H	. . . . . I . . . . G M . . S . . . . V A . . . T . I T . . . D . . . . D . . D . E . N . F K Y S .	1523
n=4		
P.hylobati	. . . . G M . . . . . H H . . . . . V T . L . T . I T . . . D . . F . . . . G . . . F . . T . F K Y S .	1505
P.inui A.hackeri	. . . . . I . . . . G M . . . . K . . . . V T . L . T . I T . . . D . . F . . . . A V . . F . . N . F K Y A .	1606
n=13	R G I S	
P.fieldi N-3	. . . . . I . . . . G . . K . . . R . . E . . . V T . . . T . I I . . . D . . . . D . . . . . . N . F K Y P A	1514
n=4	R D N T E N S	
P.vivax Sal-1	. D . . . L . . . . G M . . . . V . . . . T . I T . . . D . . . . E . . . . . . N . F K Y S S	1432
n=43	P	
P.cynomolgi Smithonian	. D . . . I . . . . G M . . . . V . . L . T . I I . . . D . . . . D . . . . . . N . . K Y S .	1467
n=10	E F	

Species	Sequence	Position
P.gonderi	S G D Y I I K D P Y K L L N L E K K K K L L G S Y K Y I D G S I D K D M A T A K D G L E Y F E K M T K L Y K E H L V S V	1465
P.fragile NIH	. . E . . M . . . . D Y . Q . . . . S A . . . . . I . . . . A . . N . . S E . . . N . M D A .	1579
n=2		
P.coatneyi	. . E . . . . D F . Q . . . . N . . S T . . . . N E . M . . Y K . . G E . . . Q . . D T .	1686
P.knowlesi H	. . E . . . . D . . Q . . . . Q . . G A . . V . . . L I . . . . . M . . Y K . . G E . . . K . . E A .	1583
n=4	N Q	
P.hylobati	T . E N . . . . D . . . . . . M . . G . . . V . . T . . E Q . L . . . N . . M A . Y N . . S D . . . K Q F D A .	1565
P.inui A.hackeri	T . . N . . . . P . . . . . D L . . . . . M . . G . N . . G M . . E . . L D . A N . G . A . Y N . . N D . . . K Q . D A .	1666
n=13	I S F R T T V A E E	
	D K A	
P.fieldi N-3	. G E . . V . . . Y . . . . D L . . . . G A . . . . . M V . . N . . . A . Y N . . G D . . . K . . D A .	1574
n=4	D D F F L A E T	
P.vivax Sal-1	. . E . . . . D . . . . I . . . . G A . . . M . L . . . N . . V T . Y N . . G E . . . T . . D G .	1492
n=43	L K V A E T A	
P.cynomolgi Smithonian	. . E . . . . D . . . . G A . V D . . M V . . N . . . A . Y Q . . G D . . . K . . D E .	1527
n=10	S I E I S E F N	

Species	Sequence	Position
P.gonderi	N E E I E K N Q K E I - - - - - - - - - - - - - - - - - - D E A K K E P S D T N P S T K V Q E L E K Y I P F L N S I K N	1507
P.fragile NIH	H A H . Q S I E N D D - S . T D G D Q L N A . N E . A K . . L . . . . Q K	1617
n=2	K G	
P.coatneyi	. . K . K E I E T S - A A S E N N Q V D A Q K E . . K . . L . . . . Q K	1723
P.knowlesi H	. A Q . K E I E A S - V P S G Q S Q L N A E K E . . K . . L . . . . Q K	1620
n=4	E Q	
P.hylobati	. . K . K D L E D Q . K E Q E E E I K I R Q E E I Q R T S N D T N E T D E I N Q L I A Q K E . S K . . L . . . . K	1625
P.inui A.hackeri	. K K . K E M E D E . K - - - - - - - - - - - - - - K I P . G E P N S A T N N Q L I A V K E E S K G . L . . . . K	1712
n=13	V D I K I M Q L N K	
	L K S	
	R	
	T	
P.fieldi N-3	. A Q . K E V . D N . N K Q D E - - - - - - - - E I K K L G T G . S N N - Q N N Q F S I . K A . . E . . L . . . S . . Q K	1626
n=4	K L K L N A E Q N	
P.vivax Sal-1	K T E I K . V E D D . K K Q D E - - - - - - - - E L K K L G N V N S Q D S K K N E F I A . K A . . . . . L . . . . . . L Q K	1545
n=43	N E Q V A E T Q S M	
	A A K L T	
	N	
P.cynomolgi Smithonian	. A Q . K E V E A N . N K H D E - - - - - - - - E I K K I G S . . S . A N D K N Q L N A . K E . . Q . . L . . . S . . Q K	1580
n=10	E H Q	
	E	

Species	Sequence	Position
P.gonderi	A Y E A L L N K V N N Y T Y N L K T V N N N L Q L E K N R T E I L I R K L D D Y T K M D E K L E D F K K Q K K E I N A E	1567
P.fragile NIH	E . . T . M . . . H I . . D . . . K F M . K Y P I . . K E . . . I V N . . E N . . . . I . . Q S . . . T D V R	1677
n=2		
P.coatneyi	E . Q S . V . T . . T . . E . . . K L M . . Y . I . . K E . D V I V K . . E Y . . . . G . . . S S . . N D V K	1783
P.knowlesi H	E . . S . V . M A H T . K E . . . K F I . . C . I . . K E . . . I V K . . E . . . . I . . N . . I Y . . S . . . S D V R	1680
n=4		
P.hylobati	E . . S . V S M A T T . . D . . . K F I . . C . I . . R E . . . I L K . . E . . I . . . . L Y R E S . . D T D V I	1685

P.inui A.hackeri<br>n=13	E . . T . V S M . T T . . N . . . K F I . . C . I . . R E . . . I . K . . E . . N . . . E K . . N Y R Q S . E E A N V I<br>S N F K R S N T K E Q K D V<br>R H	1772
P.fieldi N-3<br>n=4	E . G S . V S . . H S . . E . . . K F L . . C E I . . K E T D . I . K . . E . Y S . M . . K . . V Y . R S . . . T D V R<br>N G D S Q N E C I N L S N I	1686
P.vivax Sal-1<br>n=43	E . . S . V S . . . T . . D . . . K . I N . C . . . . K E A . . T V K . . Q . . N . . . . E Y . . S E . K N E V K<br>S	1605
P.cynomolgi Smithonian<br>n=10	E . S T . V . . . H S . . D T . . K I I . . C . I . . K E . E T I V N . . E . . S . . . . E . D V Y . K S . . . D D V K<br>N A Q E N F L Y D I K N K I Q N L R<br>G	1640

P.gonderi	H - D L L K K L K V S E V M K G N E S K A I L S E L L N V N T T L L N M D S E H K C I D T V V P E N A A C Y R Y L D G R	1626
P.fragile NIH<br>n=2	S S G . . E . F . S . K L I N E E . . . K V . . K . . . . Q . Q . . H L S . D . . . I . . . . . D . . . . T<br>I T	1737
P.coatneyi	S S G . . E . F . N . K L I N E E . . . K V . . Q . . . M Q . K M . . . G . . . . T . . . . T	1843
P.knowlesi H<br>n=4	S G . . E . . K N . K L I N E E . . . K V . . Q . . . . Q . Q M . . . S . A . . . . . . N . . . . T<br>R	1740
P.hylobati	S S V . . E . . . K . K F I N E E . . . K . . . . V . K M E . Y . . . I G T G . . . . . . Q	1745
P.inui A.hackeri<br>n=13	S S G . . D . . . K . K L I N E E . . . K . . . . V . . M G . H . . . . G . E . . . . . . T . . D . . . . L . . .<br>V Y D R F	1832
P.fieldi N-3<br>n=4	S S G . . E . . . N . K L I N E E . . . K V . . . . Q . Q M . . . G . . . . N . . E . . . . T<br>T T N H Q	1746
P.vivax Sal-1<br>n=43	S S G . . E K . M K . K L I . E . . . . E . . . Q . . . . Q . Q . . T . S . . . T . . . . N . . D . . . . T<br>R	1665
P.cynomolgi Smithonian<br>n=10	S S G . . E . . M N . K L I N Q E . . . K A . . . . Q . Q M . . . S . . . R . . . . N . . . . T<br>K E V G K	1700

P.gonderi	E E W R C L L K F K L D G G K C V A A T D V T C E D N N G G C A P D A E C K Q M D N K D I V C K C T N E G S Q P L F D G	1686
P.fragile NIH<br>n=2	. . . . T . . E V S . . . . P . P N M . . M . . . . E . . . S M P . . . E . . . . . . R . . . E . . . Q .<br>K	1797
P.coatneyi	. . . . N . . E L E . . . I P . P . M . . N E . . . . E . . . . M T E S . K . . . . . . K . . . E	1903
P.knowlesi H<br>n=4	. . . . G . . E V . . . . . P . S - I . . . E . . . . E . . . T M D . K . E V E . . . . K . . . E . . . E .	1799
P.hylobati	. . . . . K . N . . K E . D . . . P . P N M . . S . . . . E . . . N M N E . - K V . . T . . K . . . E . . . E .	1804
P.inui A.hackeri<br>n=13	. . . . N . . K E . D . . . A . S N . . . T . . . . E . . . N M N E . N Q V . . . . . K . . . E . . Y E .<br>Q E E D<br>K	1892
P.fieldi N-3<br>n=4	. . . . N F K E E G . . . . P . P . M . . K . . . . E . . . . M N E . . E . . . . . . K . . . E . . . Q .<br>Y R A D E	1806
P.vivax Sal-1<br>n=43	. . . . T . . E E . . . . . P . S N . . . K . . . . E . . . . M T . S N K . . . . . . K . . . E . . . E .<br>E	1725
P.cynomolgi Smithonian<br>n=10	. . . . Y F . E . A . . . . P . P N M . . K . K . . . . E . . . . M N D K N E . . . . . . K . . . E . . . Q .<br>N Y G N E S	1760

P.gonderi	V F C S S S S F L S L S F L L L I L F I L L C S E L	1712
P.fragile NIH<br>n=2	. . . . L F . . . M . .	1823
P.coatneyi	. . . . L F . . . M . .	1929
P.knowlesi H<br>n=4	. . . . I F F . S M . .	1825
P.hylobati	. . . . L A L F . . . M . .	1830
P.inui A.hackeri<br>n=13	. . . . S . . . . L . . L F L F . . . M . .<br>Y F L L	1918
P.fieldi N-3<br>n=4	. . . . I . L L . . . M . -<br>M	1831
P.vivax Sal-1<br>n=43	. . . . M . L F . . . M . .	1751
P.cynomolgi Smithonian<br>n=10	. . . . L F . . . M . .	1786

Additional figure S1: Amino acid sequence alignment of MSP-1 from *P. vivax* and *P. vivax*-related simian malaria parasite species. Sequences were divided into 9 blocks according to sequence similarity: 5 interspecies conserved blocks and 4 variable blocks (gray-shaded). Excluding short unaligned regions (blue-shaded), 4176 bp in the 5 conserved blocks were used for constructing phylogenetic trees and inferring evolutionary analyses. Borders between the 5'- and central regions and between the central- and 3'-regions are marked with vertical red lines. Amino acid sites under selection as detected by the omegaMap (Wilson and McVean 2006) are red-boxed for *P. vivax, P. inui* and *P. cynomolgi*, and those as detected by the HyPhy/Datamonkey (Kosakovsky Pond and Frost 2005) are pink-or beige-shared. For *P. vivax*, 5 computations were done to detect positively selected sites using HyPhy/Datamonkey for 14 randomly selected msp1 sequences from 43 sequences, due to a technical reason inherent to the program. Positively selected sites repeatedly detected 3 times or more are pink-shaded and those less than 2 are beige-shaded.
