# Supplementary material for: Lineage-specific positive selection at the merozoite surface protein 1 (msp1) locus of Plasmodium vivax and related simian malaria parasites
Source: BMC Evol Biol. 2010 Feb 19;10:52. doi: 10.1186/1471-2148-10-52 (PMC2832629; doi:10.1186/1471-2148-10-52)
Supplement: Additional file 4 — Phylogenetic trees for estimating divergence times among P. vivax and P. vivax-related simian malaria parasite lineages. Figure S2 showing a method for phylogeny-based estimation of divergence times among P. vivax and P. vivax-related simian malaria parasite lineages. [file 1471-2148-10-52-S4.PDF]

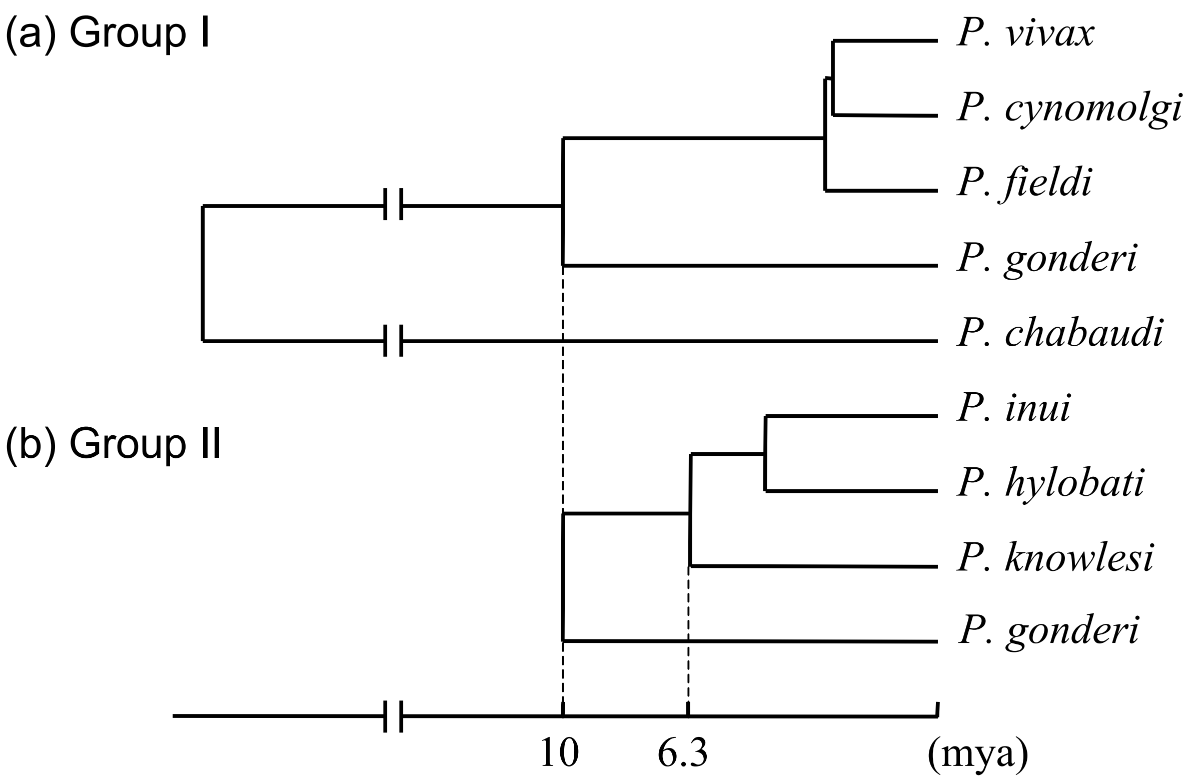

Additional figure S2. Phylogenetic trees for estimating divergence times among *P. vivax* and *P. vivax*-related simian malaria parasite lineages. Two trees were separately constructed for Group I (a) using *P. chabaudi* as an outgroup, and Group II (b) using *P. gonderi* as an outgroup because of evolutionary rate heterogeneity between the two groups. Tree topologies were extracted from fig. 1a. Branch lengths correspond to genetic distances. Calculations for divergence time were performed by adopting 10 million years ago for the divergence between *P. gonderi* and Asian malaria parasites and 6.3 million years ago for the divergence between *P. knowlesi* and *P. inui*/*P. hylobati* . Sequences of parasite strains used for each species are the same as used in Figure 2.
