# Supplementary material for: Lineage-specific positive selection at the merozoite surface protein 1 (msp1) locus of Plasmodium vivax and related simian malaria parasites
Source: BMC Evol Biol. 2010 Feb 19;10:52. doi: 10.1186/1471-2148-10-52 (PMC2832629; doi:10.1186/1471-2148-10-52)
Supplement: Additional file 5 — Positional overlap of positively selected amino acid regions of MSP-1 from P. vivax, P. inui and P. cynomolgi. Figure S3 showing five overlapping positively selected amino acid sequence regions of P. vivax, P. inui and P. cynomolgi that were inferred by the omegaMap. [file 1471-2148-10-52-S5.PDF]

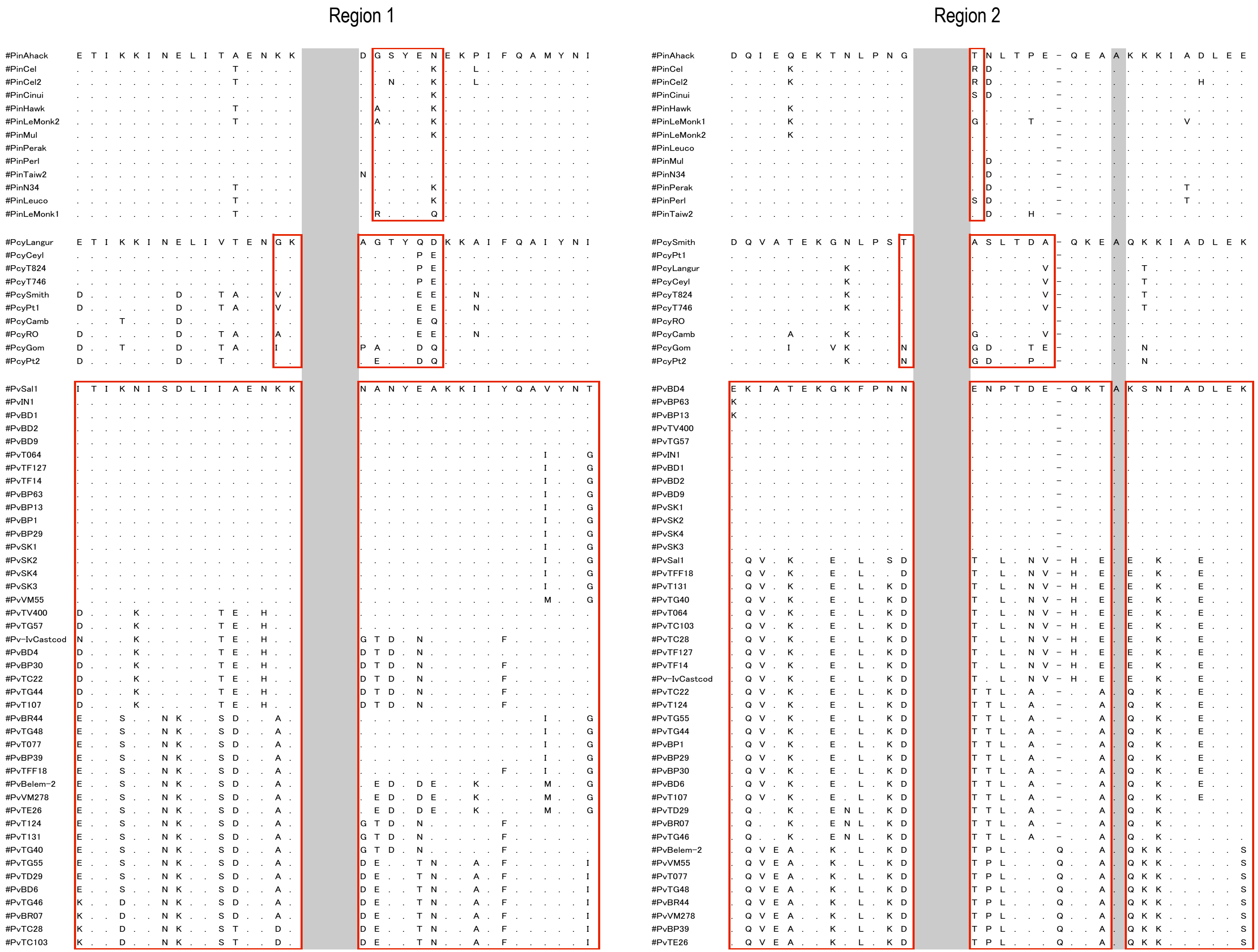

Additional figure S3. Positional overlap of positively selected amino acid regions of MSP-1 from *P. vivax*, *P. inui* and *P. cynomolgi*. Five positively selected sequence regions of *P. vivax* MSP-1 (region 1 to region 5), inferred by the omegaMap (Figure 3), are shown together with corresponding sequences of *P. inui* and *P. cynomolgi*. Regions in red boxes correspond to those in Additional figure 1. Gray shaded regions are those of short unaligned regions containing indels and repeats.

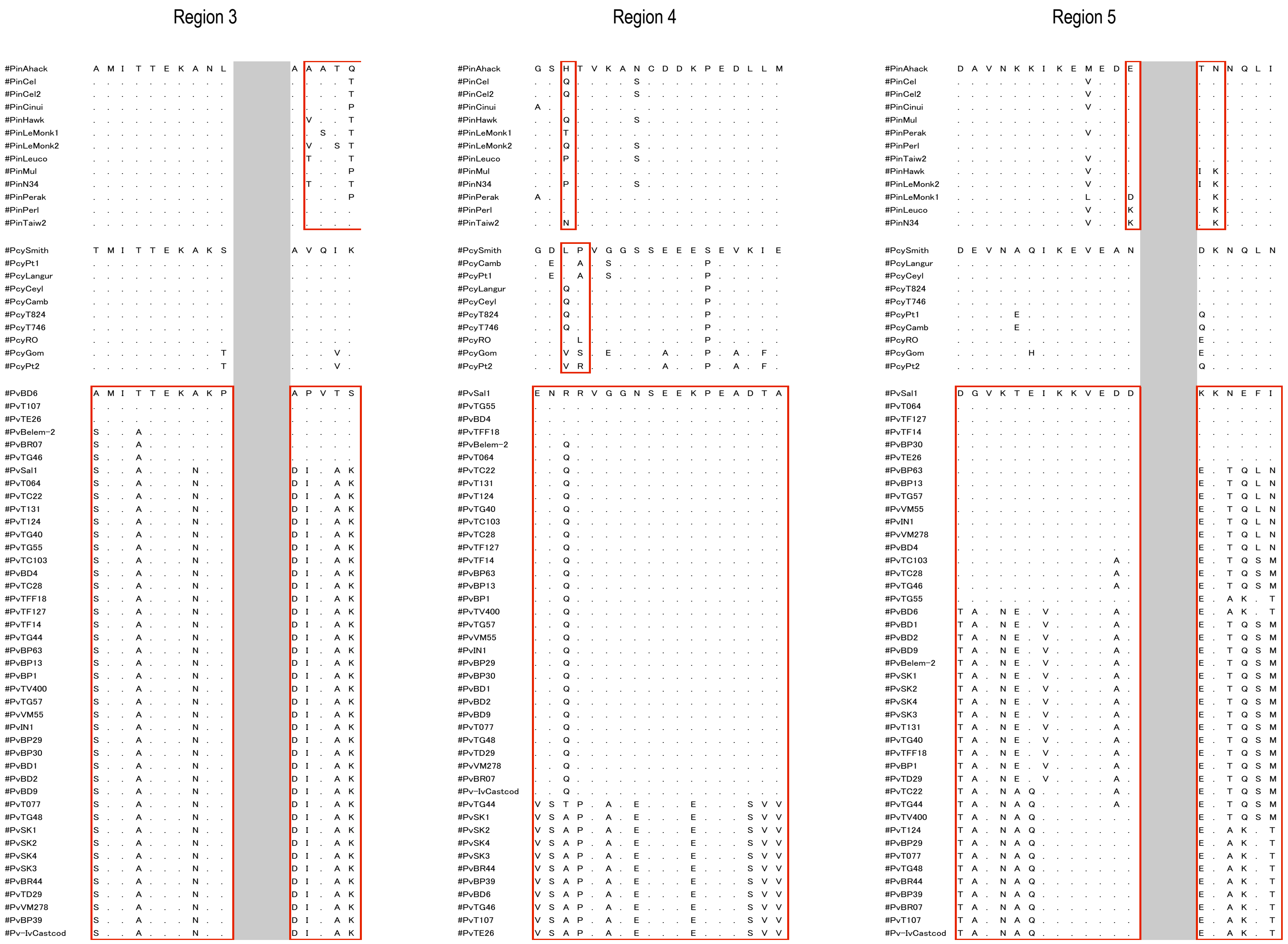

Additional figure S3 (continued)
