# Supplementary material for: Lineage-specific positive selection at the merozoite surface protein 1 (msp1) locus of Plasmodium vivax and related simian malaria parasites
Source: BMC Evol Biol. 2010 Feb 19;10:52. doi: 10.1186/1471-2148-10-52 (PMC2832629; doi:10.1186/1471-2148-10-52)
Supplement: Additional file 6 — Indels and repeats in a variable block (VB4) of msp1 from P. inui. Figure S4 showing a highly variable sequences due to indels and repetitive sequences in a variable block (VB4) of P. inui msp1. [file 1471-2148-10-52-S6.PDF]

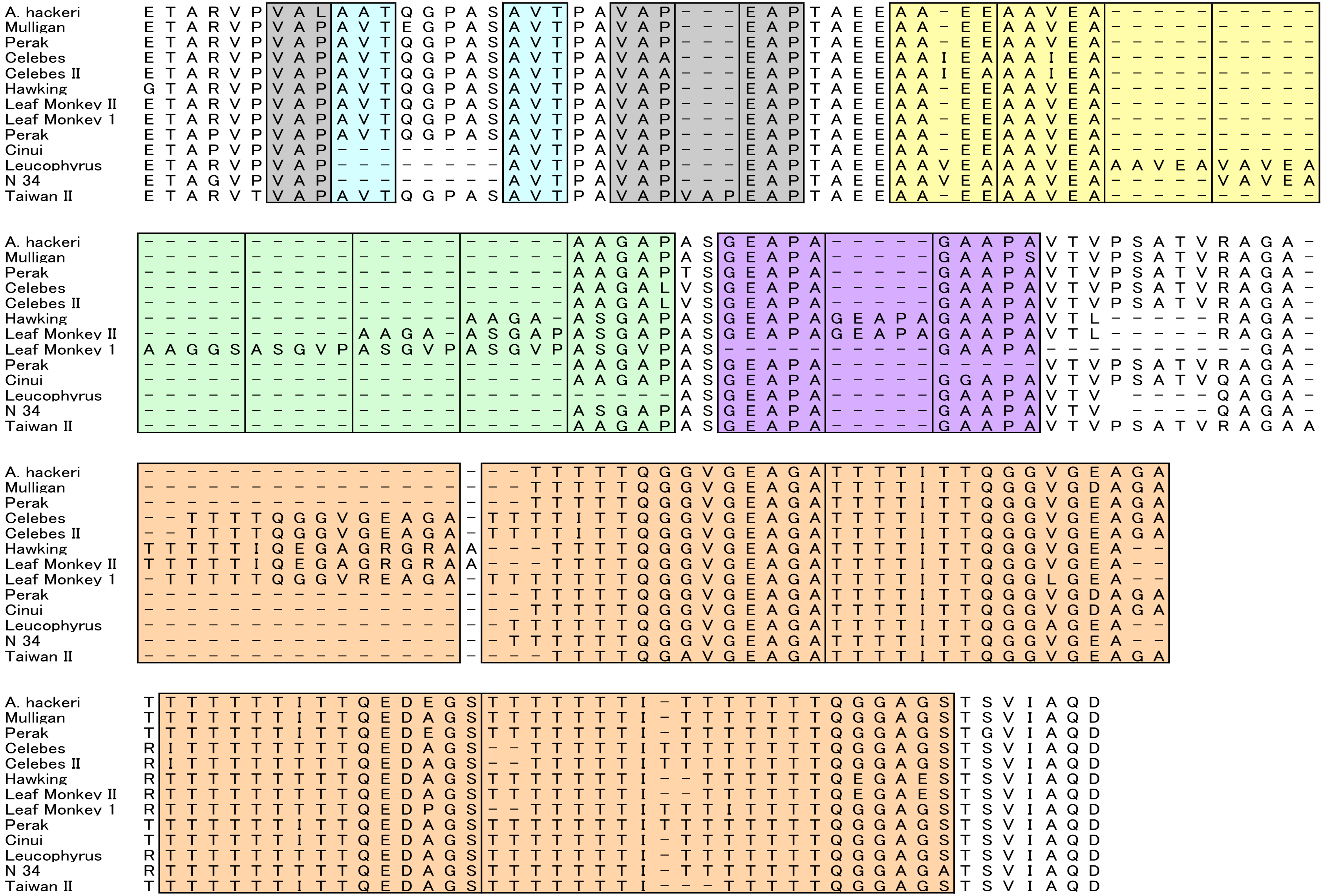

Additional figure S4. Indels and repeats in a variable block (VB4) of *msp1* from *P. inui*. Various repeating units are shown by different colors. Dashes denote deletions.
